# Cell death analysis of inducible, titratable neurodegenerative disease models in zebrafish and human stem cell-derived retinal organoids

**DOI:** 10.1242/dmm.052747

**Published:** 2026-07-24

**Authors:** Anneliese Ceisel, Gianna Graziano, Kevin Emmerich, Xiangqian Shi, Tae-In Kam, Miguel Flores-Bellver, M. Natalia Vergara, Silvia Aparicio-Domingo, Boris M. Brenerman, Anne Vielle, Elsie M. Williams, Abigail V. Sharrock, Uche Onuchuwku, Georgina S. Martinez, Lydia G. Sanders, Uzoamaka Nwagbo, Beichen Wang, Huanhuan Xiao, Grant Kroeschell, Yiqi Gao, Daniel J. Choe, Caroline E. Clouatre, Diego Alfaro Carcoba, Barak Reibman, Catalina Rodriguez, Kevin Yang, Shreya Banerjee, Frazer Matthews, James H. Thierer, Genevieve Stein-O'Brien, Ted M. Dawson, Valina L. Dawson, David F. Ackerley, M. Valeria Canto-Soler, Liyun Zhang, Jeff S. Mumm

**Affiliations:** ^1^ Wilmer Eye Institute, Johns Hopkins School of Medicine, Baltimore, MD 21231, USA; ^2^ Translational Vascular Medicine Branch, National Heart, Lung and Blood Institute, National Institutes of Health, Bethesda, MD 20814, USA; ^3^ Department of Pharmaceutical Sciences, University of Illinois-Chicago, Chicago, IL 60612, USA; ^4^ Neuroregeneration and Stem Cell Programs, Institute for Cell Engineering, Johns Hopkins University School of Medicine, Baltimore, MD 21205, USA; ^5^ Department of Neurology, Johns Hopkins University School of Medicine, Baltimore, MD 21205, USA; ^6^ CellSight Ocular Stem Cell and Regeneration Research Program, Department of Ophthalmology, Sue Anschutz-Rodgers Eye Center, University of Colorado Anschutz, Aurora, CO 80045, USA; ^7^ Solomon H. Snyder Department of Neuroscience, Johns Hopkins University School of Medicine, Baltimore, MD 21205, USA; ^8^ School of Biological Sciences, Victoria University of Wellington. Wellington 6140, New Zealand; ^9^ Te Matapihipihi - The Centre for Biodiscovery and Maurice Wilkins Centre for Molecular Biodiscovery, Victoria University of Wellington, Wellington 6140, New Zealand; ^10^ Department of Ophthalmology, University of Pittsburgh, Pittsburgh, PA 15219, USA; ^11^ Department of Physiology, Pharmacology and Therapeutics, Johns Hopkins University School of Medicine, Baltimore, MD 21205, USA

**Keywords:** Cell death, Nitroreductase, Parthanatos, Inducible disease model, Neurodegeneration, Retinal organoids

## Abstract

Inducible disease models enable large-scale screening by providing control over pathology onset, such as cell death in neurodegenerative disease. The nitroreductase (NTR)/prodrug system of cell ablation has facilitated investigations of cell function and regeneration but has not been widely adopted as a disease modeling platform, perhaps owing to assumptions that the cell death mechanism(s) elicited is artificial in nature. Prior reports suggested that NTR/prodrug-mediated death occurred through apoptosis, necroptosis and/or parthanatos, which have all been implicated in neurodegenerative disease. To clarify this issue, we investigated the cell death pathway(s) elicited by the prodrug metronidazole (MTZ) with improved nitroreductase enzyme variants. We assessed the cell death pathway(s) elicited by NTR 2.0-expressing zebrafish retinal neurons using a transcriptomic analysis, chemical inhibitors and gene-targeting assays. NTR-H, a novel NTR variant, was tested – and found to be effective – in human stem cell-derived retinal organoids. Parthanatos was implicated across all conditions tested, while evidence of apoptosis was variable. As parthanatos is associated with neurodegeneration, our results support the use of the NTR/MTZ system to create inducible neurodegenerative models targeted to specific disease-relevant neuronal cell types.

## Teaser

**Editor's choice:** Disease-relevant cell death pathways are elicited by the nitroreductase/metronidazole-based cell ablation system, suggesting potential utility as an inducible, titratable neurodegenerative disease modeling platform amenable to large-scale screening.

## INTRODUCTION

Neurodegenerative disease affects millions of people worldwide. The incidence of dementia in the developed world alone was estimated at 13.5 million in 2000 and is projected to increase to 36.7 million in 2050 (Zheng and Chen, 2022). With respect to eye disease, the number of people with age-related macular degeneration was estimated at 196 million in 2020 and is projected to increase to 288 million in 2040 (Wong et al., 2014). As the average age of the global population continues to increase, neurodegenerative diseases will become pervasive (Gammon, 2014; Zheng and Chen, 2022). Unfortunately, available treatments are limited in efficacy and typically designed to treat symptoms rather than prevent neurodegeneration or, better yet, restore lost neural functions (Zheng and Chen, 2022). Therefore, it is crucial to generate models of neurodegenerative disease that enable the interrogation of disease mechanisms as well as the discovery and development of next-generation neuroprotective and neuroregenerative therapeutics.

Established mutational genetic animal models of Alzheimer's disease (AD), Parkinson's disease (PD), retinitis pigmentosa (RP), age-related macular degeneration (AMD), glaucoma and many more neurodegenerative diseases have yielded insights into mechanisms of neurodegeneration and potential targets for pan-disease and disease-specific treatments (Fletcher et al., 2011; Inoue et al., 2021; Niwa et al., 2016; Pennesi et al., 2012; Tu et al., 2015; Zhang et al., 2020, 2021). However, mutant models can be difficult to apply to large-scale drug discovery, or even lead candidate validation, owing to phenotypic variation, i.e. incomplete penetrance and/or variability in the onset of neuronal cell loss. Although variability does typically reflect the reality of complex disease states, such variation also necessitates untenable sample sizes regarding large-scale preclinical animal model testing. Inducible models of neurodegenerative disease can address this issue by providing control over a key disease phenotype: the onset of neuronal cell loss.

Inducible neurodegenerative models have been developed using heritable and non-heritable approaches. Non-heritable models use a variety of means to damage and/or eliminate disease-associated cells, including chemical toxins [e.g. N-methyl-D-aspartate (NMDA), aminoglycosides, endoplasmic reticulum stressors (tunicamycin, thapsigargin), etc.] or physical damage (light ablation, optic nerve crush, etc.) (Hernández et al., 2007; Le Bras, 2022; Luo et al., 2019; Niwa et al., 2016; Sharrock et al., 2022; Sonntag et al., 2012; White and Mumm, 2013). These inducible neurodegenerative models have been used to reveal fundamental mechanisms of degeneration, such as excitotoxicity (Mohd Sairazi et al., 2015; Olney, 1969), roles of neighboring glia (Myer et al., 2006) and the immune system (Wu et al., 2002). They have also provided mechanistic insight into neuronal regeneration, such as radial glia driving adult central nervous system regeneration (Kroehne et al., 2011) or Muller glia as the source of new neurons in the regenerating zebrafish retina (Fausett and Goldman, 2006). Nevertheless, these models typically do not accurately emulate disease phenotypes owing to the induction of rapid and widespread cell loss. For example, tunicamycin-induced endoplasmic reticulum stress has been advanced as a model of diabetic retinopathy, AMD, glaucoma and RP. However, tunicamycin treatments typically induce loss of cone and rod photoreceptors as well as retinal capillary degeneration in mice and rats (Shirai et al., 2015; Wang et al., 2019) – effects that may not manifest in every disease this drug is used to model. Similarly, another method used to generate non-heritable retinal degeneration models – light ablation – can lead to retinal cell loss across all three retinal cell layers (Lyu et al., 2023). Although non-heritable inducible models of degenerative diseases can be convenient, bypassing the need to develop genetic tools, they often elicit cell death in a manner inconsistent with disease progression.

Heritable inducible degeneration models can overcome some of these limitations by allowing control over the extent, rate and/or duration of cell loss, and by targeting disease-linked cell types directly. These models use transgenic expression of an effector protein that, when combined with a ligand/substrate, causes death of the transgene-targeted cell type (Sharrock et al., 2022). In mice, targeted ablation of horizontal cells was achieved by expressing the diphtheria toxin (DT) receptor behind the connexin 57 promoter and intraperitoneally injecting DT (Sonntag et al., 2012). More recently, selective ablation of rod photoreceptors, retinal ganglion cells (RGCs) and other cell types has been achieved by generating transgenic animals (e.g. zebrafish, medaka, frog and jellyfish) that express a nitroreductase (NTR) enzyme under control of a cell type-specific promoter (Curado et al., 2007; D'Orazi et al., 2020; Fogerty et al., 2022; Hall et al., 2022; Huang et al., 2013; Leach et al., 2022; Mazaheri et al., 2014; Montgomery et al., 2010; Pisharath et al., 2007; Sharrock et al., 2022; Tabor et al., 2014; Weissbourd et al., 2021; White and Mumm, 2013; Zhang et al., 2021). When the prodrug metronidazole (MTZ) is administered, NTR enzymes convert it to a cell-entrapped cytotoxin, killing the NTR-expressing cells (Bridgewater et al., 1997; Lewis, 2013; Sharrock et al., 2022). The NTR/MTZ system has been used to study the physiological roles of cells and investigate mechanisms regulating cellular regeneration in regenerative species such as zebrafish (Chen et al., 2023; Curado et al., 2008; Emmerich et al., 2023, 2024; Fraser et al., 2013; Huang et al., 2013; Leach et al., 2022; Pisharath et al., 2007; White et al., 2017; White and Mumm, 2013) and frogs (Martinez-De Luna and Zuber, 2018). Although the NTR/MTZ system is useful for physiologically mimicking disease-relevant cell loss, its relevance as a degenerative disease modeling platform remains unclear owing to a lack of understanding about which cell death pathways are triggered in response to NTR/MTZ-induced DNA damage and, in particular, whether all cell types respond equivalently.

Historically, neurodegenerative diseases have been linked to apoptosis owing to a binary view of cell death, i.e. that cells die either by damage-associated necrosis or genetically programmed apoptosis (Fink and Cookson, 2005). However, the number of genetically mediated cell death ‘subroutines’ has expanded dramatically in recent years (Galluzzi et al., 2012, 2018), leading to growing appreciation that neurodegeneration may be mediated by one or more alternative cell death pathways. Indeed, increasing evidence now implicates a variety of cell death pathways – e.g. necroptosis, ferroptosis, parthanatos, etc. – in neurodegenerative diseases (Jiang et al., 2021; Moujalled et al., 2021; Zhang et al., 2017). For example, parthanatos, or poly ADP-ribose (PAR)-dependent cell death, has been strongly implicated in PD (Hu et al., 2021; Lee et al., 2014), as well as photoreceptor dystrophies (Sahaboglu et al., 2010) and may be involved in neurodegeneration more broadly (Park et al., 2020). Ideally, the specific type of cell death attending a given disease should be recapitulated in the models used to understand the disease.

Here, we investigated the type(s) of cell death induced by the NTR/MTZ system in zebrafish and in human stem cell (HSC)-derived retinal organoids as a means of assessing relevance for neurodegenerative disease modeling. The first-generation NTR enzyme, referred to here as ‘NTR 1.0’, had previously been associated with apoptosis (Chen et al., 2011; Curado et al., 2007; White and Mumm, 2013). However, our recent analysis suggests that NTR 1.0/MTZ cell ablation is mediated by several DNA damage-associated cell death pathways implicated in neurodegenerative diseases, including apoptosis, necroptosis and parthanatos (Galluzzi et al., 2012, 2018; Zhang et al., 2021). With the advent of new NTR variants (Guise et al., 2007; Rich et al., 2023; Sharrock et al., 2022) and alternative prodrugs (Bergemann et al., 2018; Chen et al., 2023; Lai et al., 2021), the mechanism(s) of cell death induced by each NTR/prodrug pairing will need to be assessed to determine relevance to disease. As an entry point, we investigated the cell death pathway(s) triggered by the conversion of the prodrug MTZ by two recently identified NTR variants: (1) a rationally engineered NTR derived from *Vibrio vulnificus*, termed ‘NTR 2.0’, which exhibits ∼100-fold improved efficacy in eliciting MTZ-induced cell ablation in zebrafish (Sharrock et al., 2022); and (2) a new NTR variant, termed ‘NTR-H’, that is effective in eliciting cell death in human cells. Notably, NTR-H could potentially be useful for the development of new models of neurodegenerative disease using HSC-derived brain and retinal organoids. For NTR 2.0/MTZ assays in zebrafish, cell death was investigated for two disease-associated retinal neuron types, rod photoreceptors and RGCs. The assays used include chemical inhibitors and CRISPR/Cas9-based knockdown of key components of multiple disease-relevant cell death pathways (Lin et al., 2025; Wu et al., 2018). For NTR-H/MTZ assays, cell death was assessed more broadly in ‘retinal neurons’ of HSC-derived retinal organoids using biochemical analyses of cell death pathway markers. We found that both NTR 2.0/MTZ and NTR-H/MTZ-induced cell ablation are associated with cell death pathways relevant to neurodegenerative disease, including parthanatos and apoptosis. Interestingly, our data suggest that parthanatos is the primary cell death pathway elicited by NTR/MTZ. We conclude that the NTR/prodrug-based cell ablation system can be used as an inducible and titratable neurodegenerative disease modeling system. However, the specific mechanism(s) of cell death elicited should be confirmed for each NTR variant/prodrug pairing and per the cell type targeted to assess relevance to the disease being modeled.

## RESULTS

The reduction of prodrugs by NTR has been shown to induce DNA damage and subsequent cell death (Chen et al., 2011; Curado et al., 2007; White and Mumm, 2013; Williams et al., 2015), as seen by the loss of cell markers (Sharrock et al., 2022; Zhang et al., 2021), terminal deoxynucleotidyl transferase dUTP nick end labeling (TUNEL) staining (Curado et al., 2007; Emmerich et al., 2023) and reduction in nuclei (Emmerich et al., 2024). NTR/MTZ-induced cell ablation was initially linked to apoptotic cell death by investigators using TUNEL and activated Caspase-3 immunostaining (Curado et al., 2007; Pisharath et al., 2007). However, these studies have major caveats, such as TUNEL not distinguishing apoptosis from other types of cell death (Ansari et al., 1993; Charriaut-Marlangue and Ben-Ari, 1995; Chen et al., 2011; de Torres et al., 1997; Dmitrieva and Burg, 2007; Kanoh et al., 1999; Grasl-Kraupp et al., 1995), the evidence for NTR/MTZ-induced activation of Caspase-3 being predominantly qualitative (Curado et al., 2007) and the discovery of anastasis (Tang et al., 2012). Since these initial studies, the cell death landscape has expanded to include many variant forms that can be distinguished experimentally (Galluzzi et al., 2018). For example, we recently implicated PARP-dependent cell death (parthanatos) and, to a lesser degree necroptosis, in NTR 1.0/MTZ-mediated ablation of rod photoreceptors (Zhang et al., 2021). Finally, new NTR variants, ‘NTR 2.0’ (Sharrock et al., 2022) and an ‘NTR-H’ variant we report here, have yet to be characterized regarding the type(s) of cell death elicited by MTZ conversion. To address these issues, we have investigated the mechanism(s) of NTR 2.0/MTZ- and NTR-H/MTZ-induced cell death from the perspective of several targeted neuronal cell types.

### Cell death signatures in transcriptomic datasets of NTR 2.0/MTZ-based ablation

**Fig. 1.**
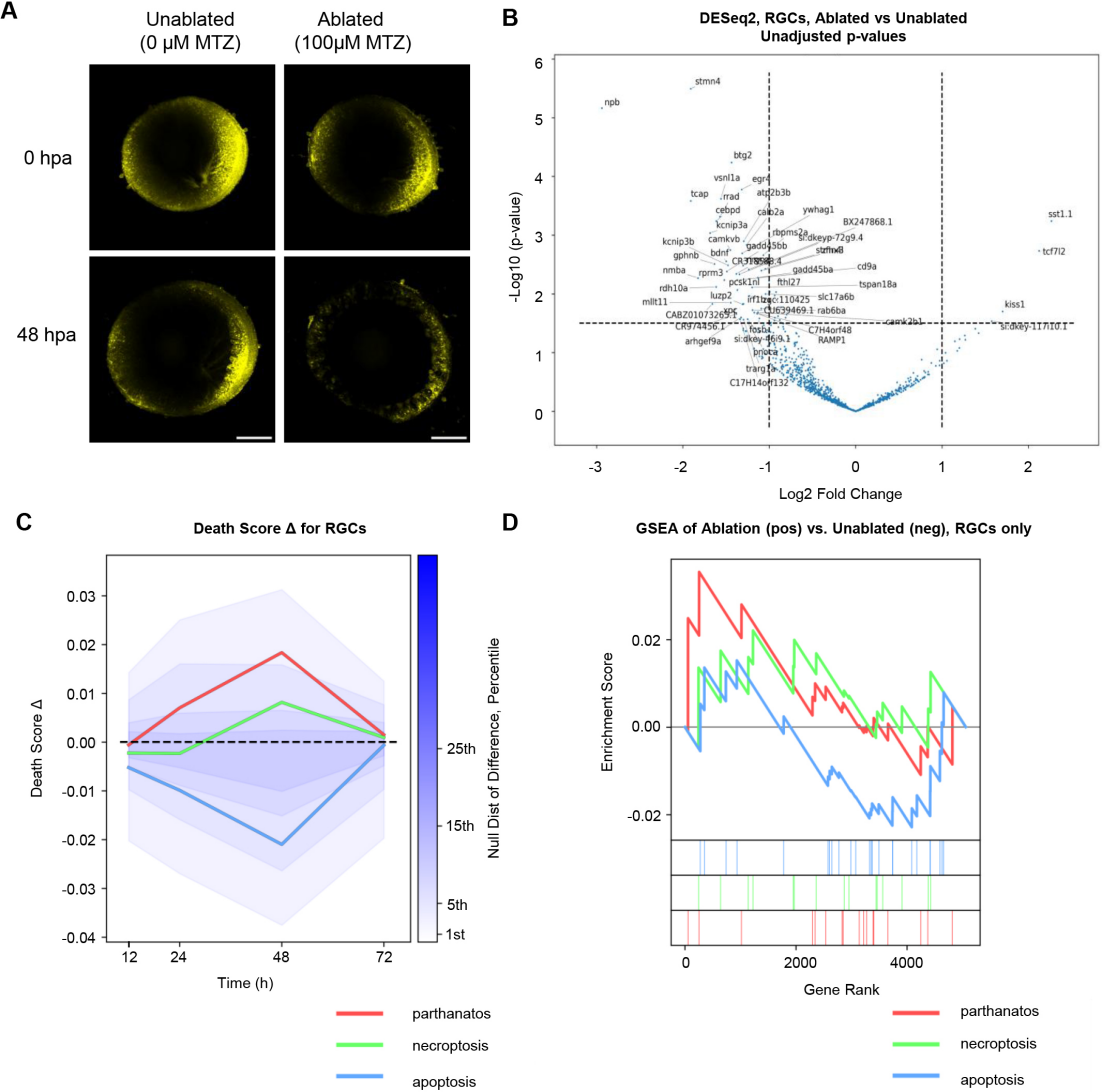
**Transcriptomic assessment of cell death in retinal ganglion cell (RGC) nitroreductase (NTR) 2.0-YFP ablation.** (A) Confocal image of a maximum-projection stack of a lateral view of NTR 2.0-based ablation of RGCs in the zebrafish eye at 0 h post ablation (hpa) and 48 hpa. Scale bars: 50 μm. (B) Volcano plot showing the fold change and unadjusted *P*-value of top differentially expressed genes (DEGs) of ablated RGCs with respect to unablated RGCs. Generated with DESeq2. (C) Difference in cell death pathway scoring of parthanatos, necroptosis and apoptosis in ablated versus unablated RGCs over time. The *y*-axis is the difference in score calculated by summing the DEGs for all genes in a cell death signature. Positive direction indicates a higher signature in the ablated condition. Bold line is the median with a bootstrap confidence interval denotation. (D) Gene set enrichment analysis for parthanatos (red), necroptosis (green) and apoptosis (blue) in ablated versus unablated RGCs. Positive direction indicates higher enrichment in the ablated condition.

To gain initial insight into the cell death pathway elicited by NTR 2.0 expressing neurons, we assessed transcriptomic signatures from an existing dataset for NTR2.0-mediated RGC ablation (GSE268179; Emmerich et al., 2024). In this single-cell RNA-sequencing (scRNA-seq) dataset, unablated versus ablated larval zebrafish eyes underwent sequencing at 12, 24, 48 and 72 h post 100 μM MTZ ablation onset (Fig. 1A). We identified the RGCs using Leiden-based clustering, cell type markers and top loadings for each cluster (Dataset 3, Fig. S1A-C). To confirm the loss of RGCs, we previously counted nuclei in the RGC layer and additionally showed that the number of differentially expressed genes (DEGs) across time goes down for RGCs in this dataset (Emmerich et al., 2024).

We next asked whether cell death pathways previously implicated in NTR1.0/MTZ ablation were enriched in the RGC NTR 2.0 ablation paradigm. To do so, we used weighted DEGs (Fig. 1A; Dataset 4) to score cell death pathways. A combination of publications was used to generate a consensus gene list/signature associated with each cell death pathway (Dataset 5) and corresponding orthologs identified in the zebrafish genome. Gene set scoring was then used to assess levels of pathway-specific expression in unablated versus ablated RGCs across time (Fig. S2A). The difference between ablated and unablated RGCs was then calculated and background confidence intervals generated by bootstrapping sham signatures of comparable size (Fig. 1B). The parthanatos-related gene signature showed the greatest change between ablated and unablated RGCs (Fig. 1B). To better understand how this pattern arose, we looked at how strongly each parthanatos gene was expressed in RGCs over time (Fig. S2B). To compliment the targeted analysis, we performed gene set enrichment analysis (GSEA) for ablated versus unablated RGCs across all normalized differential expression values and scanned the Kyoto Encyclopedia of Genes and Genomes (KEGG) and Wikipathways signature sets, finding enrichment in some immune-related terms (phagosome and Il-6 signaling pathway; Fig. S2C). When assessing parthanatos, necroptosis and apoptosis specifically, we detected parthanatos-enriched pathways (Fig. 1C), which corroborated the proposed mechanism we previously observed with NTR 1.0/MTZ-mediated ablation of rod photoreceptors. Taken together, these results indicated that, of the pathways assessed, the primary mechanism of cell death from NTR2.0-expressing RGCs is parthanatos.

### NTR 2.0/MTZ-mediated ablation of rod photoreceptors

**Fig. 2.**
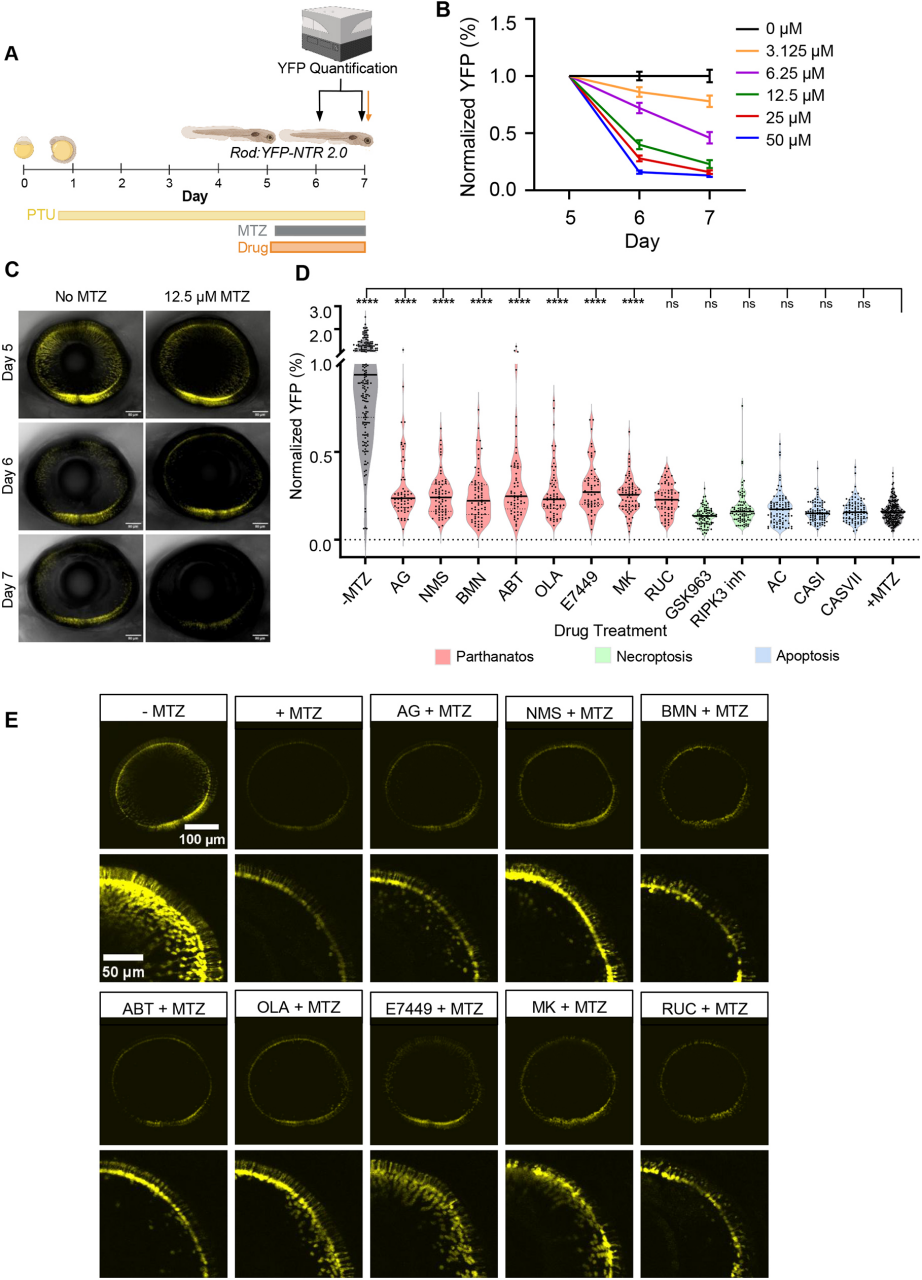
**Chemical inhibition of NTR 2.0-expressing rods.** (A) Schematic depicting the timeline of MTZ treatment to study cell death in *rod:YFP-NTR 2.0* larvae as well as the timeline of drug treatment for D and E. Created in BioRender by Mumm, J. (2026). https://BioRender.com/jlvzpn5. This figure was sublicensed under CC-BY 4.0 terms. (B) MTZ titration response curve showing fluorescence levels of rods from 5 to 7 days post fertilization (dpf) following MTZ treatment with six different concentrations of MTZ across three experimental replicates. All data are normalized to the same-day control. Mean±s.e.m. displayed. *N*=40-42 fish per condition (0 μM, 40 fish; 3.125 μM, 41 fish; 6.25 μM, 40 fish; 12.5 μM, 41 fish; 25 μM, 42 fish; 50 μM, 41 fish; all biological replicates. Data for each condition were collected across three repeated experiments. Mean, s.d., *N* and *P*-values can be found in Datasets 6 and 7. (C) Representative *in vivo* confocal microscopy showing *rod:YFP-NTR 2.0* larvae pre- and post-ablation with 12.5 μM MTZ. Scale bars: 50 μm. (D) Fluorescence quantification across three experimental replicates following rod cell ablation and treatment with various cell death pathway chemical inhibitors. Red plots designate inhibition of the parthanatos pathway, green plots designate necroptosis, and blue plots indicate apoptosis. All fish are normalized to the no MTZ control. One-way Kruskal–Wallis performed with Dunnett's post-hoc analysis. Black line is the median. *N*=61-211 fish per condition (no MTZ, 188 fish; AG, 61 fish; NMS, 70 fish; BMN, 71 fish; ABT, 62 fish; OLA, 62 fish; E7449, 61 fish; MK, 71 fish; RUC, 71 fish; GSK963, 94 fish; RIPK3 inh, 89 fish; AC, 88 fish; CASI, 89 fish; CASVII, 90 fish; +MTZ, 211 fish; all biological replicates). Data for each condition were collected across three repeated experiments. Mean, s.d., *N* and *P*-values can be found in Datasets 6 and 7. (E) Representative confocal images and an inset of 7 dpf rod control fish (−MTZ; +MTZ) and fish treated with chemical inhibitors. YFP indicates rod photoreceptors. The intensity of YFP was increased in the insets to resolve morphological detail of the rods. Scale bars: 100 μm. ns, not significant; ****P≤0.0001. Each dot represents an individual larval fish. ABT, veliparib; AC, Ac-DEVD-CHO; AG, AG-14361; BMN, talazoparib; CASI, Caspase-3/7 inhibitor I; CASVII, Caspase-3 inhibitor VII; MK, niraparib; MTZ, metronidazole; NMS, NMS-P118; OLA, olaparib; PTU, N-phenylthiourea; RIPK3, receptor-interacting protein kinase 3 inhibitor; RUC, rucaparib.

To facilitate a direct comparison to our prior biological characterization of NTR 1.0/MTZ-induced rod cell death (Zhang et al., 2021), we next investigated cell death mechanisms induced by MTZ treatment of NTR 2.0-expressing rod photoreceptors. We previously confirmed rod cell loss through manually quantifying the loss of rhodopsin antibody staining (Sharrock et al., 2022). Thus, we first established a titrated cell death assay by exposing 5 day post-fertilization (dpf) transgenic larvae co-expressing NTR 2.0 and yellow fluorescent protein (YFP) in rod photoreceptors – *Tg(rho:GAP-YFP-2A-nfsB_Vv F70A/F108Y)jh405*, hereafter *rod:YFP-NTR 2.0* (Fig. 2A) – to a range of MTZ concentrations (0, 3.125, 6.25, 12.5, 25 and 50 μM; Fig. 2B; Dataset 6). YFP expression – as a proxy for rod photoreceptor changes – was quantified before and at 1 and 2 days post-MTZ exposure using an established fluorescence plate reader assay (Walker et al., 2012; White et al., 2017). YFP levels of MTZ-treated larvae were normalized to same-day no MTZ controls and plotted across all three time points to assess rod cell death kinetics and to compare the extent of cell loss between titrated MTZ exposures. The data showed that the 12.5 μM concentration resulted in a relatively slow and linear loss of rod cells over the 2 days of MTZ treatment, while still achieving maximal cell loss at 7 dpf (∼80%; Fig. 2B; Dataset 6). Confocal *in vivo* imaging confirmed that YFP levels in the retina were significantly lower in MTZ-treated retinas than in unablated controls (Fig. 2C). The 12.5 μM MTZ treatment was chosen as a sensitized assay, i.e. slow steady cell loss paradigm, for all subsequent experiments used to assess potential modulators of rod cell death kinetics in *rod:YFP-NTR 2.0* larvae at 6 and/or 7 dpf.

### Chemical and genetic inhibition of NTR 2.0/MTZ-mediated ablation of rod photoreceptors

Previously, we used chemical inhibitors of different cell death pathways to assess how rod cells expressing NTR 1.0 die when exposed to MTZ (Zhang et al., 2021). The results showed that inhibitors of parthanatos and necroptosis promoted rod cell survival whereas inhibitors of apoptosis did not. Here, to investigate how rod cells expressing NTR 2.0 die when exposed to MTZ, we again tested chemical inhibitors of parthanatos, necroptosis and apoptosis. In total, eight inhibitors of parthanatos, two of necroptosis and three of apoptosis were tested (Dataset 1) using concentrations previously established for NTR 1.0-expressing rod cells (Zhang et al., 2021). At 5 dpf, *rod:YFP-NTR 2.0* larvae were pre-treated with individual chemical inhibitors for 4 h followed by addition of MTZ, creating a 12.5 μM MTZ and 1× inhibitor treatment for another 48 h. Rod cell survival was then quantified using the plate reader assay at 7 dpf (Fig. 2A). Seven of eight parthanatos inhibitors promoted rod cell survival to statistically significant levels, with protective effects ranging from 9% to 20% (Fig. 2D; Dataset 6). Confocal images confirmed an increased number of rod photoreceptors in retinas treated with parthanatos inhibitors than in retinas treated with MTZ only (Fig. 2E). However, inhibitors of both necroptosis and apoptosis failed to show any neuroprotective effect (Fig. 2D,E; Dataset 6). These data suggest that MTZ treatment of NTR 2.0-expressing rods elicits a Parp-dependent cell death pathway, such as parthanatos or cGMP-dependent cell death (Tolone et al., 2019).

**Fig. 3.**
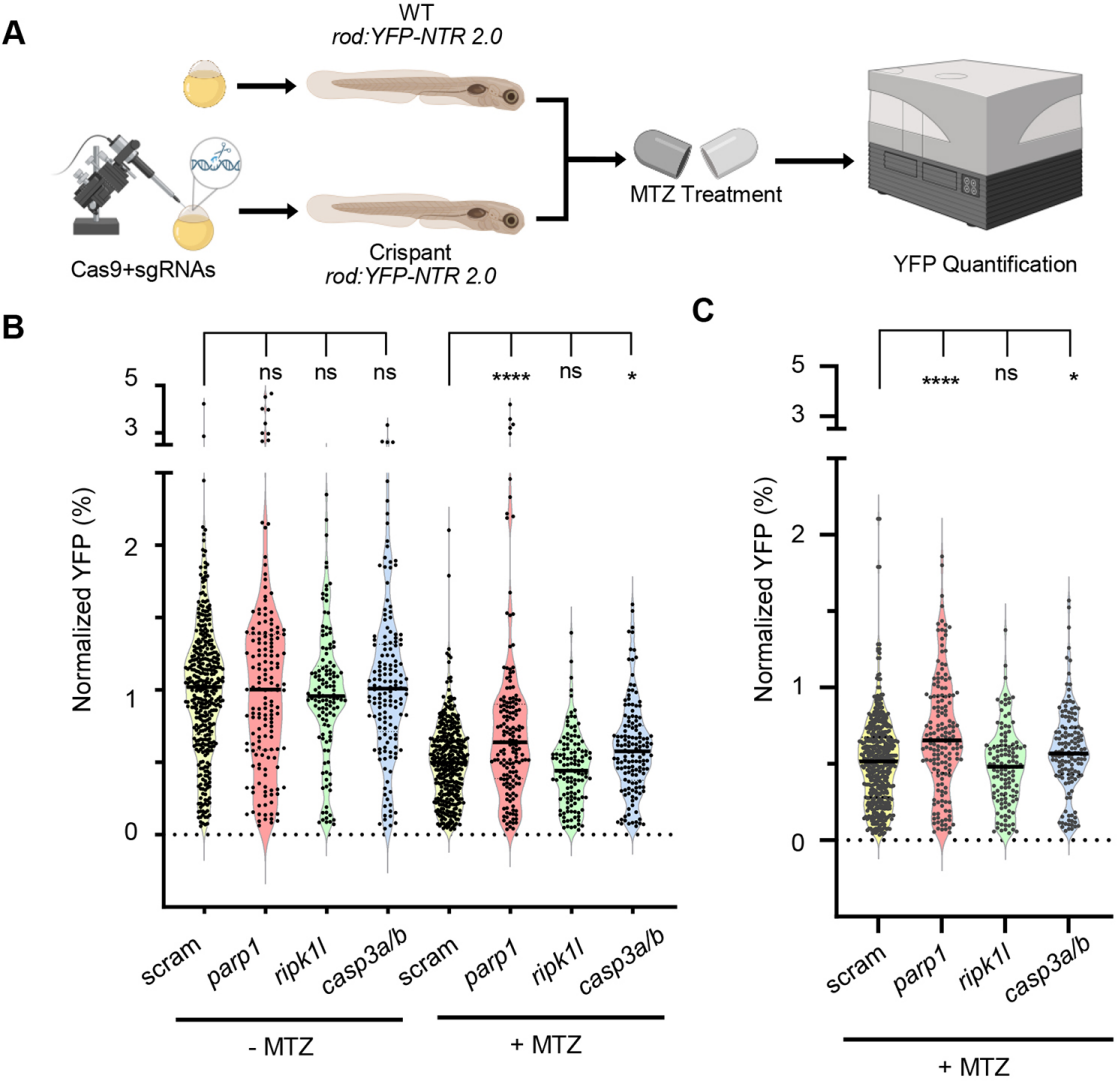
**Cell death induced by MTZ treatment of NTR 2.0-expressing rod cells – crispants.** (A) Schematic demonstrating the crispant assay workflow in *rod:YFP-NTR 2.0* larvae. Created in BioRender by BioRender. Mumm, J. (2026). https://BioRender.com/phqfzak. This figure was sublicensed under CC-BY 4.0 terms. (B) Effect of 2× sgRNA redundant gene knockdowns on rod cell numbers, obtained via fluorescence quantification, when fish were treated ±MTZ for 24 h. Crispants were normalized to the scrambled no MTZ control. *N*=3. One-way Kruskal–Wallis performed with Dunnett's post-hoc analysis. (C) Cell death response of rods in 2× sgRNA redundant targeting crispants following 24 h treatment with MTZ, each normalized to their respective no MTZ crispant control. One-way Kruskal–Wallis performed with Dunnett's post-hoc analysis. ns, not significant; *P≤0.05; ****P≤0.0001. Each dot represents an individual larval fish. Gene knockdowns are *parp1* (red), *ripk1l* (green) and *casp3a/b* (blue). For all violin plots, the black line is the median. *N*=124-373 fish per condition (no MTZ *scram*, 373 fish; no MTZ *parp1*, 159 fish; no MTZ *ripk1l*, 124 fish; no MTZ *casp3a/b*, 149 fish; +MTZ *scram*, 360 fish; +MTZ parp1, 160 fish; +MTZ *ripk1l*, 124 fish; +MTZ *casp3a/b*, 142 fish). All fish are biological replicates. Data for each condition were collected across three repeated experiments. Tables summarizing *N*, average and *P*-values can be found in Datasets 6 and 7.

To further interrogate cell death pathways mediating MTZ-induced ablation of NTR 2.0-expressing rod cells, we performed a genetic knockdown analysis. Using a previously established CRISPR/Cas9-based method of creating G0 mutants (i.e. crispants; Lin et al., 2025; Wu et al., 2018), we targeted key genes in each cell death pathway: *parp1* for PARP-dependent cell death (e.g. parthanatos), *ripk1l* for necroptosis and *casp3a*/*casp3b* for apoptosis (Fig. 3A). Two single-guide RNAs (sgRNAs) targeting each key gene, or scrambled sgRNA controls, were co-injected with Cas9 protein into one-cell-stage embryos. Quantitative PCR confirmed the reduced expression of each target gene (Fig. S3A,B, Dataset 8). Larvae were then treated ±12.5 μM MTZ at 5 dpf for 24 h. YFP levels were quantified at 6 dpf by plate reader assay.

To account for effects on rod development, we first asked whether *parp1*, *ripk1l* and *casp3a/b* crispants had altered rod cell numbers. Plate reader assays showed no statistically significant differences in YFP levels across all conditions at 6 dpf, suggesting no change in rod genesis rates in crispant larvae (Fig. 3B). However, crispants exposed to 24 h MTZ treatment to induce rod loss found YFP levels were elevated to 76% in *parp1* crispants and 60% in *casp3a/b* crispants relative to the 50% in scrambled controls, suggesting neuroprotective effects. Conversely, *ripk1l* crispants showed no evidence of neuroprotective effects (Fig. 3B; Dataset 6). To control for any developmental effects, all 6 dpf MTZ-treated values were normalized respective to each crispants' developmental effect (Fig. 3C). Again, *parp1* and *casp3a/b* crispants showed evidence of rod neuroprotection (68% and 58%, respectively, versus 50% in scrambled sgRNA controls). To ascertain whether the effects were attributable to off-target sgRNA activity, we repeated these assays using a new set of four sgRNAs per target. Developmentally, we saw no statistically significant increase in rod-based YFP amongst groups (Fig. S2C, Dataset 6). Again, quantitative PCR confirmed the reduced expression of each targeted gene (Fig. S3C, Dataset 9). In the presence of MTZ, knockdown of *parp1* and *casp3a/*b resulted in increased rod cells (69% and 72%, respectively) whereas *ripk1l* did not (39%, Fig. S3D), akin to our two-sgRNA tests. Normalization to each crispants' no MTZ YFP levels to fully account for any developmental effect confirmed that knockdown of *parp1* and *casp3a/b* resulted in a neuroprotective effect (41% and 33% reduction in YFP loss, respectively; Fig. S3E, Dataset 6). Collectively, these findings are consistent with our transcriptomic analysis (Fig. 1) and prior investigation of cell death mechanisms underlying MTZ-induced cell death of NTR 1.0-expressing rod cells (Zhang et al., 2021), suggesting that exposing NTR 2.0-expressing rods to MTZ elicits a largely conserved cell death response.

### NTR/MTZ-mediated ablation of RGCs

**Fig. 4.**
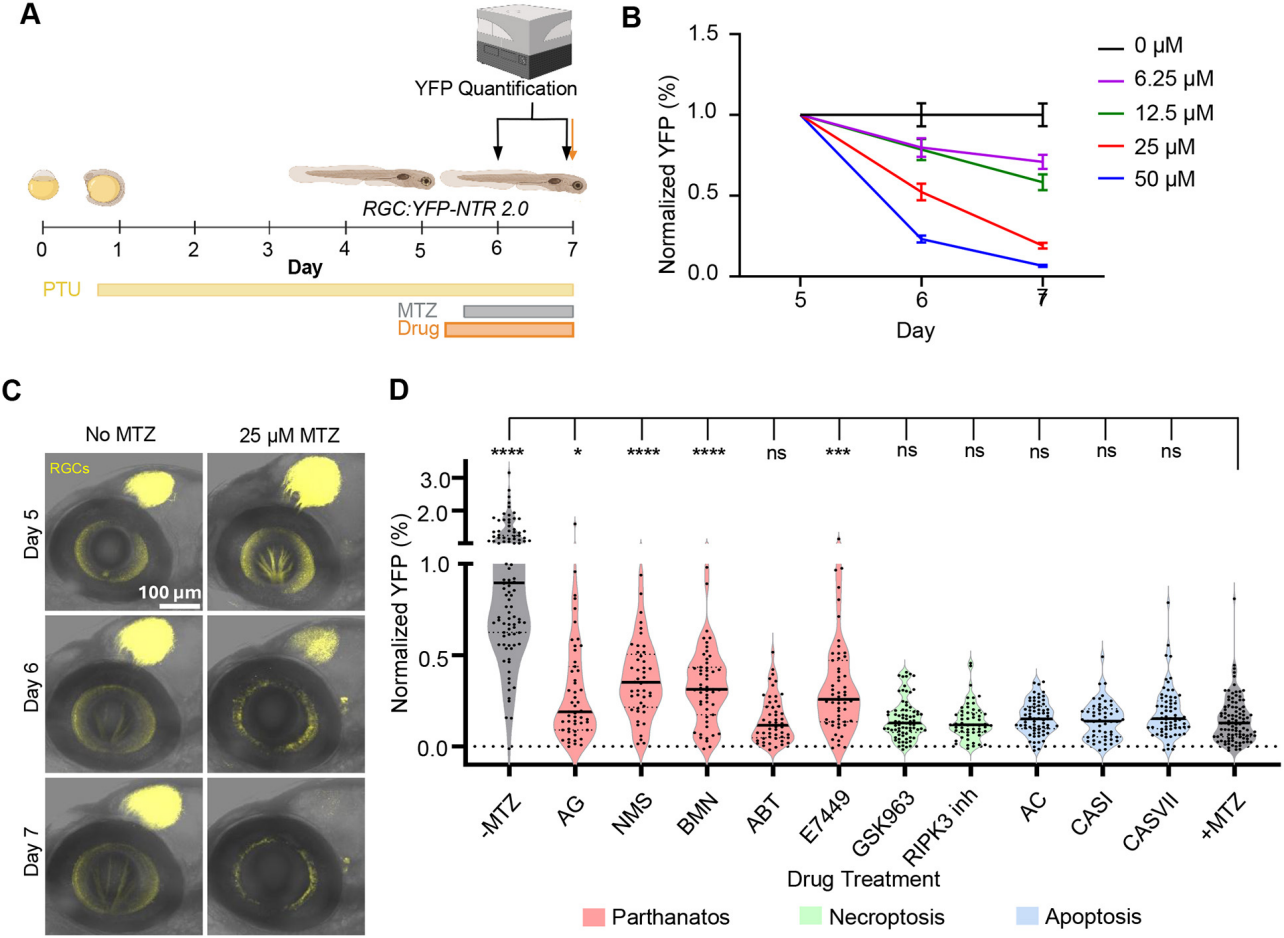
**Cell death induced by MTZ treatment of NTR 2.0-expressing RGCs – inhibitors.** (A) Schematic depicting the timeline of MTZ treatment to study cell death in *RGC:YFP-NTR 2.0* larvae. Created in BioRender by Mumm, J. (2026). https://BioRender.com/7tnh9j1. This figure was sublicensed under CC-BY 4.0 terms. (B) MTZ titration response curve showing fluorescence levels of RGCs from 5 to 7 dpf following MTZ treatment with five different concentrations of MTZ. All data are normalized to the same-day control. Mean±s.e.m. displayed. *N*=57-69 fish per condition (0 μM, 60 fish; 6.25 μM, 57 fish; 12.5 μM, 60 fish; 25 μM, 69 fish; 50 μM, 66 fish; all biological replicates). Data for each condition were collected across three repeated experiments. Mean, s.d., *N* and *P*-values can be found in Datasets 6 and 7. (C) Representative *in vivo* confocal microscopy showing *RGC:YFP-NTR 2.0* larvae pre- and post-ablation with 25 μM MTZ. Scale bar: 100 μm. (D) Fluorescence quantification across three experimental replicates following RGC cell ablation and treatment with various cell death pathway chemical inhibitors. Red plots designate inhibition of the parthanatos pathway, green plots designate necroptosis, and blue plots indicate apoptosis. *N*=51-100 fish per condition (no MTZ, 98 fish; AG, 51 fish; NMS, 45 fish; BMN, 51 fish; ABT, 51 fish; E7449, 55 fish; GSK963, 76 fish; RIPK3 inh, 55 fish; AC, 69 fish; CASI, 53 fish; CASVII, 68 fish; +MTZ, 100 fish; all biological replicates). Data for each condition were collected across three repeated experiments. Mean, s.d., *N*, effect (%), fold change and *P*-values can be found in Datasets 6 and 7. One-way Kruskal–Wallis performed with Dunnett's post-hoc analysis. ns, not significant; *P≤0.05; ***P≤0.001; ****P≤0.0001. Each dot represents an individual larval fish. For all violin plots, the black line is the median. Tables summarizing *N*, average and *P*-values can be found in Datasets 6 and 7.

Thus far, our *in vivo* analysis of NTR/MTZ-induced cell death has been limited to rod photoreceptor cells. Whether other cell types undergo the same or different forms of cell death in the context of NTR/MTZ-mediated cell ablation remains an open question. Our transcriptomic analysis in Fig. 1 suggests that the cell death pathways elicited for NTR/MTZ-induced ablation of RGCs may be similar to that for ablation of rods. To further investigate this, we evaluated cell death pathway induction in larval zebrafish with NTR2.0-YFP expressing RGCs. We generated a RGC degeneration model by crossing an *isl2b* promoter-based Gal4 driver line targeting RGCs, *Tg(isl2b:GAL4,myl7:TagRFP)zc65* (Fujimoto et al., 2011), with a UAS-based reporter/effector line facilitating co-expression of NTR 2.0 and YFP, *Tg(5xUAS:GAP-tagYFP-P2A-nfsB_Vv F70A/F108Y)jh513* (Emmerich et al., 2023; Sharrock et al., 2022; Fig. 4A; hereafter *RGC:YFP-NTR 2.0*). Similarly to in the rod cell tests, we first established a titrated RGC death assay by treating 5 dpf larvae with four different concentrations of MTZ – 6.25, 12.5, 25 and 50 μM – and quantifying YFP levels as a proxy for RGC loss at 6 and 7 dpf using our plate reader assay (Fig. 4A). All YFP values were normalized to same-day controls and plotted across time to show the kinetics of RGC death (Fig. 4B; Dataset 6). In comparison to the rod model, RGCs required a slightly higher MTZ concentration (25 μM) to reach an equivalent level of YFP loss at 6 and 7 dpf (50% and 80%, respectively; Fig. 4B). Confocal images confirmed the loss of RGCs over time during a 25 μM MTZ treatment (Fig. 4C). Akin to rod assays, the concentration of MTZ enabling a slow, steady loss and maximal ablation at 7 dpf was selected as a sensitized assay for all downstream experiments.

### Chemical and genetic inhibition of NTR 2.0/MTZ-mediated ablation of RGCs

To interrogate cell death mechanisms in NTR 2.0-expressing RGCs exposed to MTZ, we again tested multiple inhibitors of cell death – five parthanatos, two necroptosis and three apoptosis inhibitors – for the ability to promote RGC survival upon MTZ treatment. At 5 dpf, *RGC:YFP-NTR 2.0* larvae were pre-treated with individual chemical inhibitors for 4 h, followed by addition of 25 μM MTZ (Fig. 4A). At 7 dpf, YFP was quantified in MTZ-treated larvae using the plate reader assay. Necroptosis and apoptosis inhibitors had no statistically significant effects on YFP levels and, thus, RGC survival. However, four of five parthanatos inhibitors led to statistically significant increases in YFP levels, consistent with neuroprotective effects ranging from 18% to 26% (Fig. 4D; Dataset 6). These results further implicate parthanatos as the predominant mechanism promoting the death of NTR 2.0-expressing RGCs exposed to MTZ.

**Fig. 5.**
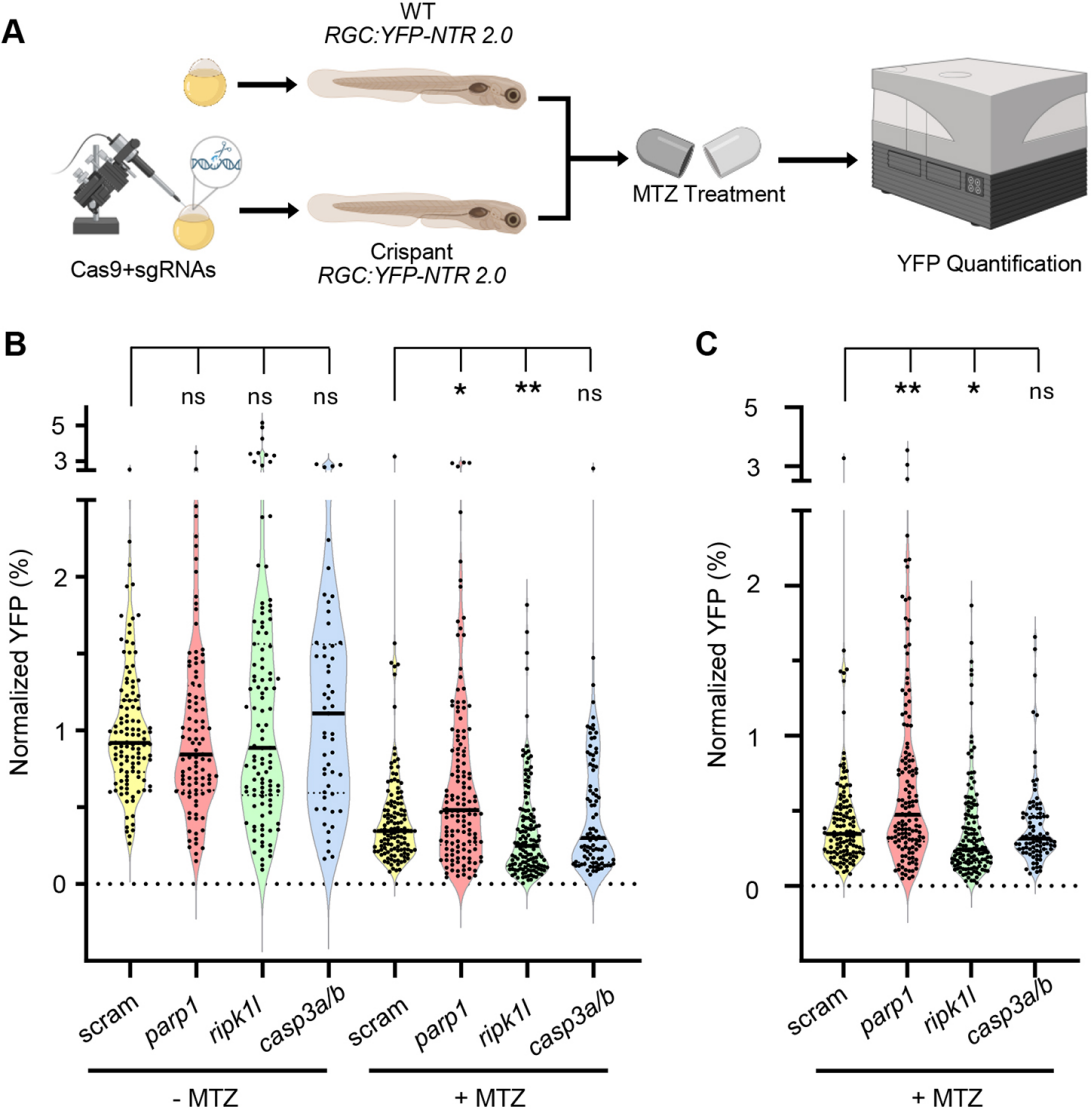
**Cell death induced by MTZ treatment of NTR 2.0-expressing RGCs – crispants.** (A) Schematic demonstrating the crispant assay workflow in *RGC:YFP-NTR 2.0* larvae. Created in BioRender by Mumm, J. (2026). https://BioRender.com/lk4qa0g. This figure was sublicensed under CC-BY 4.0 terms. (B) Effect of 2× sgRNA redundant gene knockdowns on RGC numbers, obtained via fluorescence quantification, when fish were treated ±MTZ for 24 h. Crispants were normalized to the scrambled no MTZ control. *N*=3. One-way Kruskal–Wallis performed with Dunnett's post-hoc analysis. (C) Cell death response of RGCs in 2× redundant targeting sgRNAs across three experimental replicates following 24 h treatment with MTZ, each normalized to their respective no MTZ crispant control. One-way Kruskal–Wallis performed with Dunnett's post-hoc analysis. ns, not significant; *P≤0.05; **P≤0.01. Each dot represents an individual larval fish. Gene knockdowns are *parp1*(red), *ripk1l*(green), and *casp3a/b* (blue). For all violin plots, the black line is the median. (B,C) *N*=53-138 fish per condition (no MTZ *scram*, 115 fish; no MTZ *parp1*, 99 fish; no MTZ *ripk1l*, 107 fish; no MTZ *casp3a/b*, 53 fish; +MTZ *scram*, 127 fish; +MTZ *parp1*, 138 fish; +MTZ *ripk1l*, 137 fish; +MTZ *casp3a/b*, 88 fish). All fish are biological replicates. Data for each condition were collected across three repeated experiments. Tables summarizing *N*, average and *P*-values can be found in Datasets 6 and 7.

As per the cell death gene targeting assays performed in the rod cell ablation model, we generated crispants to assess effects of disrupting discrete cell death pathways on RGC survival. Injected larvae were treated ±MTZ at 5 dpf. YFP levels were quantified by plate reader assay at 6 dpf (Fig. 5A). In the absence of MTZ, we found no difference in YFP levels amongst crispants and the scrambled control, suggesting no change in RGC genesis (Fig. 5B). In the presence of MTZ, *parp1* crispants had increased YFP levels compared to those of scrambled sgRNA control (67% versus 44%, respectively). Interestingly, we found that *ripk1l* crispants showed evidence of increased RGC loss (34% versus 44% YFP), whereas *casp3a/b* crispants had no statistically significant effect (47% versus 44% YFP, Fig. 5B). Normalizing to each crispants' no MTZ control to fully account for any developmental effects confirmed alterations in RGC numbers for *parp1* and *ripk1l* crispants (70% and 37%, respectively, compared to 45% in the scrambled sgRNA controls), as well as a lack of effect in *casp3a/b* crispants (Fig. 5C). To ascertain whether these effects were attributable to off-target activity of sgRNAs, crispant tests were repeated using a new set of four sgRNAs per target (Dataset 1). Here, we observed that *casp3a/b* knockdown led to a slight decrease in RGC genesis (Fig. S4A, Dataset 6). In MTZ-treated larvae, *parp1* crispants had a near-statistically significant increase in YFP levels relative to those of uninjected controls (64 versus 53%, a predicted 24% reduction in RGC loss), suggesting increased RGC survival (Fig. S4A, Dataset 6). Conversely, *ripk1l* and *casp3a/b* crispants showed no statistically significant effects on YFP levels at 6 dpf, suggesting no alteration in RGC survival. Again, these results were strengthened after fully accounting for any developmental effects on RGC numbers (Fig. S4B). Knockdown of *parp1* resulted in a statistically significant increase in RGCs relative to those of the controls (73% versus 52%, a 43% reduction in cell death). Meanwhile, *ripk1l* and *casp3ab* knockdown had no statistically significant effect. These results, in accordance with inhibitor assay results and our transcriptomic analysis, suggest that NTR 2.0/MTZ-induced cell death in RGCs occurs primarily through parthanatos.

### Using the NTR/MTZ system for neurodegenerative disease modeling in human induced pluripotent stem cell (hiPSC)-derived retinal organoids

**Fig. 6.**
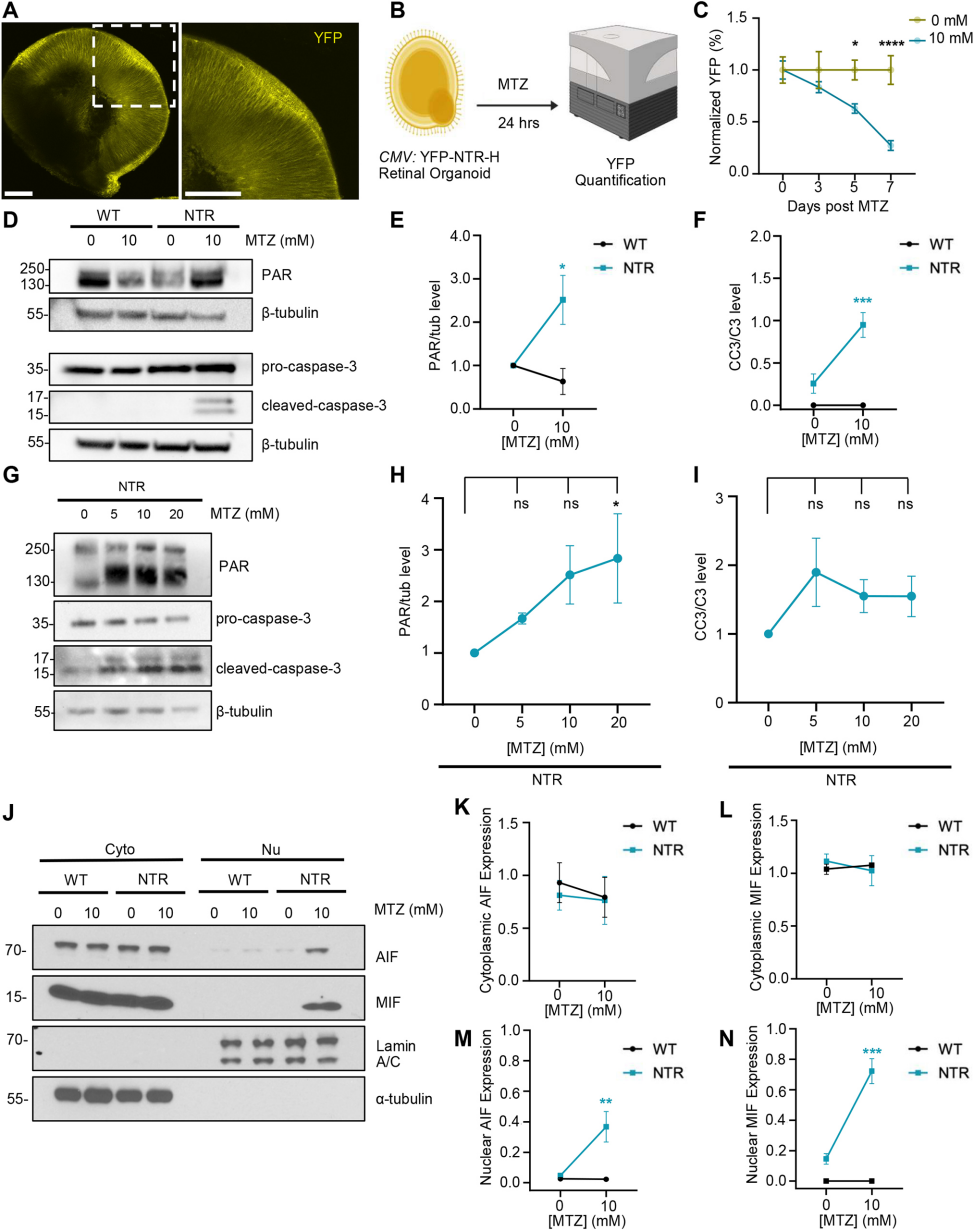
**NTR/MTZ-induced targeted cell death in transgenic YFP-NTR-H human retinal organoids.** (A) Representative confocal image of NTR-H-YFP expression in a retinal organoid at 60 days of differentiation (left) and a zoomed-in confocal image of YFP-expressing retinal organoid cells (right). Scale bars: 100 μm; 40 μm (inset). (B) Schematic depicting the experimental workflow for *CMV:YFP-NTR-H* retinal organoids. Created in BioRender by Mumm, J. (2026). https://BioRender.com/ses5ot9. This figure was sublicensed under CC-BY 4.0 terms. (C) Comparison of MTZ-induced ablation over time in *CMV:YFP-NTR-H* retinal organoids between two different MTZ concentrations. *N*=4; Mean±s.e.m. displayed. Two-way ANOVA with Šídák's multiple comparisons correction performed [*P*-value for interaction is 0.0139; for day (D)3 0 mM versus 10 mM is 0.0209; for D7 0 mM versus 10 mM is <0.0001]. Biological replicates. This experiment was repeated twice. See Dataset 10 for more details. (D-F) Representative immunoblots (D) and quantification of PAR (E) and cleaved caspase-3 (CC3)/procaspase-3 (C3) (F) levels in the wild-type (WT) and transgenic retinal organoids exposed to 10 mM MTZ for 24 h. *N*=3. Mean and s.e.m. displayed. Two-way ANOVA with uncorrected Fisher's LSD with a single pooled variance. For PAR/β-tubulin, significant *P*-values are 0.0032 for treated WT versus NTR; 0.0102 for NTR untreated versus treated; and 0.0188 for interaction. For CC3/C3, significant *P*-values are <0.0001 for treated WT versus NTR; 0.0007 for NTR untreated versus treated; and 0.0057 for interaction. See Dataset 10 for more details. (G-I) Representative immunoblots (G) and quantification of PAR (H) and cleaved CC3/C3 (I) levels in transgenic retinal organoids exposed to MTZ (0, 5, 10 and 20 mM) for 24 h. *N*=3. Mean±s.e.m. displayed. Kruskal–Wallis test with Dunnett's multiple comparisons correction to the 0 μM MTZ condition. The significant *P*-value for PAR levels is 0.0262 for 0 versus 20 μM MTZ. See Dataset 10 for more details. (J-N) Translocation of both AIF and MIF were examined by western blotting (J) of the cytosolic (K,L) and nuclear fractions (M,N) in WT and transgenic (NTR) retinal organoids exposed to 10 mM MTZ for 24 h. Equivalent protein loading was confirmed by appropriate loading markers (α-tubulin and lamin A/C). Mean±s.e.m. displayed. Two-way ANOVA with uncorrected Fisher's LSD (*N*=3). For AIF and MIF cytoplasmic fractions, there are no significant *P*-values. For AIF nuclear fractions, *P*-values are 0.0013 for treated WT versus NTR; 0.0020 for NTR untreated versus treated; and 0.0125 for interaction. For MIF nuclear fractions, *P*-values are 0.0001 for treated WT vs NTR; 0.0002 for NTR untreated versus treated; and 0.0024 for interaction. (D,G,J) Three independent experiments were performed for all treatment conditions for protein collection. A minimum of five retinal organoids per sample were collected. See Dataset 10 for more details ns, not significant; *P≤0.05; **P≤0.01; ***P≤0.001; ****P≤0.0001.

In zebrafish, the investigation of cell death mechanisms was limited to chemical inhibitors and crispant assays owing to a lack of vetted antibodies facilitating biochemical analysis. Although analyzing chemical inhibition and gene disruption can be informative of pathways involved in cell death, there is the possibility that, by disrupting a cell death mechanism, we shunt dying cells down an alternative cell death pathway and, in doing so, mask the involvement of the original pathway. To enable the evaluation of biochemical markers of cell death, and to assess whether NTR could be used to model neurodegenerative diseases in HSC-derived organoids, we used transgenic hiPSC-derived retinal organoids expressing an NTR protein. During the process of identifying NTR 2.0 as a variant providing enhanced ablation efficacy in zebrafish (Sharrock et al., 2022), we also identified an NTR variant that enabled effective ablation of hiPSCs and hiPSC-derived neurons at MTZ concentrations that do not display toxicity in non-transgenic human cells (Fig. S5A-D). We refer to this NTR variant (NfsB_Cs, which comes from the bacterial species *Cronobacter sakazakii*) as ‘NTR-H’, to denote its utility in ablating human cells. We established a hiPSC transgenic line for co-expressing NTR-H and YFP in HSCs and derived tissues, including retinas (hereafter *CMV:YFP-NTR-H*; Fig. 6A). This line was used to generate retinal organoids through a well-established differentiation protocol (Zhong et al., 2014), which allowed us to perform a comprehensive biochemical analysis of NTR/MTZ-mediated cell death in hiPSC-derived retinal neurons.

We previously adapted our plate reader assay to enable quantification of fluorescent reporter intensity in transgenic or reporter-labeled retinal organoids, a methodology we termed three-dimensional automated reporter quantification (3D-ARQ; Vergara et al., 2017). Here, we used this approach to assess reporter levels in transgenic retinal organoids co-expressing YFP and NTR-H, relative to non-transgenic wild-type (WT) and chimeric retinal organoids, to further validate the technique (Fig. S5E,F). Subsequently, we quantified MTZ-induced retinal cell loss in these organoids as assessed by loss of YFP signal (Fig. 6B,C; Dataset 10).

Having confirmed the ability of the NTR-H/MTZ system to induce cell death in hiPSC-derived retinas, we then set out to determine which cell death pathways are triggered when NTR-H-expressing cells are exposed to MTZ. A distinct advantage of performing these studies on human cells was the ability to use biochemical methods. We first asked whether MTZ treatment of YFP-NTR-H expressing organoids led to cleavage of caspase-3 to the activated form. WT (non-transgenic) and YFP-NTR-H-expressing retinal organoids were treated ±10 mM MTZ for 24 h and then harvested for western blotting with antibodies against full-length caspase-3, activated (cleaved) caspase-3 and β-tubulin (as a loading control) (Fig. 6D-F; Dataset 10). The data showed that MTZ treatment of the retinal organoids co-expressing YFP and NTR-H led to cleavage of caspase-3 to the activated form, whereas no evidence of cleavage was found in the three control conditions. To assess potential involvement of other cell death pathways, we performed a western blot assay for PAR polymer accumulation (as a marker of PARP activation and indicator of parthanatos) on retinal organoids co-expressing YFP and NTR-H. We also tested whether a lower concentration of MTZ was sufficient for induction of cell death. Control and transgenic retinal organoids were treated ±MTZ at 5, 10 and 20 mM for 24 h and then harvested for western blotting. Although significance was not reached in ImageJ analyses, blots provided qualitative evidence that both caspase-3 cleavage and PAR accumulation were evident, with PAR accumulation showing statistical significance at 20 mM MTZ (Fig. 6G-I; Dataset 10). To further interrogate the involvement of PARP-dependent cell death, WT and transgenic organoids were treated ±10 mM MTZ for 24 h. Cells were then harvested, proteins were separated into nuclear and cytoplasmic fractions, and western blotting was performed with antibodies against apoptosis-inducing factor (AIF) and macrophage migration inhibitory factory (MIF), lamin A/C (a nuclear marker) and α-tubulin (as a cytoplasmic marker; Fig. 6J-N; Dataset 10). In parthanatos, AIF is released from the mitochondria into the cytosol and is translocated into the nucleus, where it mediates large-scale DNA fragmentation and chromatin condensation (Galluzzi et al., 2018). Cytosolic AIF promotes translocation of MIF (macrophage migration inhibitory factor) into the nucleus, where MIF catalyzes DNA cleavage (Galluzzi et al., 2018). The results showed that MTZ treatment of YFP-NTR-H expressing retinal organoids led to nuclear translocation of AIF and MIF, a hallmark of induction of parthanatos (Galluzzi et al., 2018). Together, these results provide proof of principle of the utility of the NTR-H/MTZ system to effectively induce pathological forms of cell death in human neurons and, thus, to potentially model neurodegenerative diseases in HSC-derived organoids expressing NTR-H in neuronal subtypes.

Even though other forms of cell death such as necroptosis were not specifically interrogated in retinal organoids, collectively, our data from rods, RGCs and hiPSC-derived retinal organoids suggest that parthanatos is the primary cell death pathway elicited by MTZ-treatment of NTR-expressing cells.

## DISCUSSION

### NTR/MTZ-induced cell death mechanisms

**Fig. 7.**
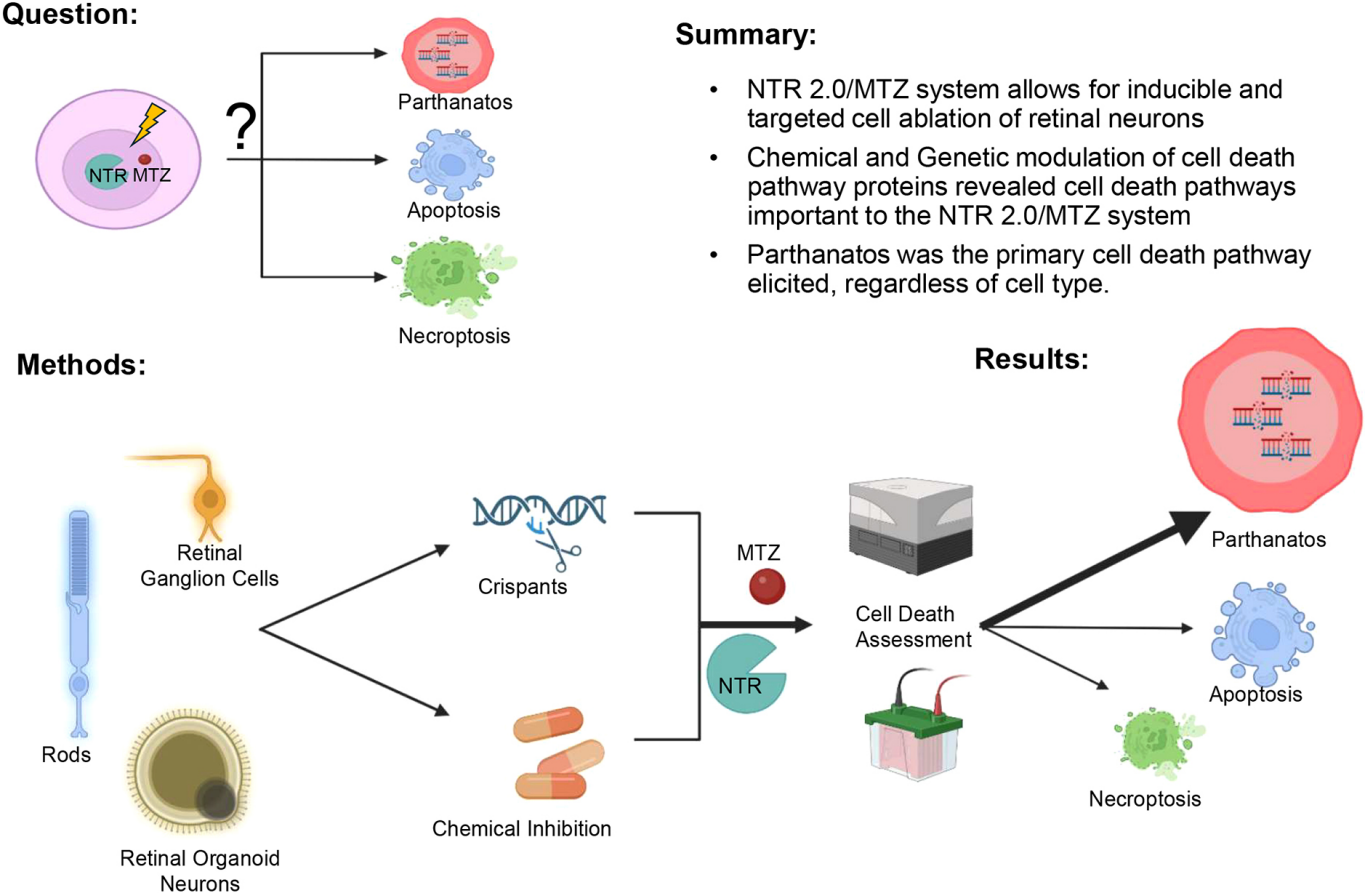
**Graphical summary.** Using genetic and chemical inhibition in cells undergoing NTR/MTZ-induced ablation, we found that, among the three pathways tested, parthanatos was the primary cell death pathway elicited. Created in BioRender by Mumm, J. (2026). https://BioRender.com/ao81qgw. This figure was sublicensed under CC-BY 4.0 terms.

In this study, we asked whether new variants of the NTR/MTZ system of targeted cell ablation, specifically NTR 2.0 and NTR-H, can be used to physiologically model diseases linked to cell loss by inducing disease-relevant cell death pathways in genetically targeted cell types (e.g. parthanatos, necroptosis and/or apoptosis; summarized in Fig. 7). Earlier studies suggested that NTR-expressing cells die by apoptosis when exposed to MTZ (Curado et al., 2007; Pisharath et al., 2007). However, more recently, we determined that NTR 1.0/MTZ-based ablation of rod photoreceptors was mediated predominantly by a DNA damage-induced cell death pathway, termed parthanatos (Zhang et al., 2021). Together, these studies have implicated apoptosis, necroptosis and parthanatos in NTR/MTZ-induced cell death. However, the first-generation NTR enzyme, *Escherichia coli* NfsB (NTR 1.0), is not ideal for modeling many diseases owing to off-target effects caused by the high concentration of prodrug required to elicit cell death (Cheng et al., 2015). We recently engineered a second-generation NTR enzyme, *V. vulnificus* NfsB F70A/F108Y (NTR 2.0), that improves prodrug-induced cell ablation efficacy ∼100-fold in zebrafish, thereby enabling prodrug treatments with lower concentrations to achieve the same or better levels of ablation (Sharrock et al., 2022). Given the different origin of the NTR2.0, we wanted to explore its neurodegenerative disease relevance. We first used a pre-existing scRNA-seq dataset to identify which of the previously implicated cell death pathways are implicated by a transcriptomic analysis of NTR2.0-mediated cell ablation. We found parthanatos to be the strongest cell death signature induced in NTR 2.0-expressing RGCs in zebrafish. However, parthanatos is primarily defined by post-transcriptional modifications and subcellular translocation of effector proteins. Such events are difficult to capture transcriptionally, which may explain the modest significance of the parthanatos signature. To validate this analysis, we used chemical inhibitors and genetic disruption assays to ask whether NTR 2.0/MTZ-induced cell death occurred by parthanatos, necroptosis or apoptosis. Collectively, the data suggest that treating NTR1- and NTR 2.0-expressing cells with MTZ elicits very similar cell death responses predominated by parthanatos. This may differ from other NTR/prodrug systems owing to the specificity of cell death. For example, the NTR/CB1954 system kills neighboring cells – effects that are leveraged for gene-directed enzyme prodrug therapy (Williams et al., 2015). Meanwhile, NTR/MTZ-based ablation results in a cell-entrapped cytotoxin with no bystander effect. We also introduce a new NTR enzyme variant that facilitates effective ablation of human cells, *Cronobacter sakazakii* NfsB (NTR-H) and interrogate the cell death pathways elicited in hiPSCs expressing NTR-H following treatment with MTZ using a biochemical analysis. We reasoned that NTR 2.0 and NTR-H may provide a means of physiologically modeling degenerative diseases in zebrafish and HSC-derived organoids, respectively, by inducing disease-relevant cell death. We found that, across all paradigms and cell death pathways tested, NTR/MTZ-elicited cell death occurs predominantly by parthanatos.

### Cell death and neurodegenerative disease models

Over the past few decades, the understanding of cell death has shifted from a morphology-based classification of apoptosis versus necrosis to one centered on essential molecular mechanistic components (Galluzzi et al., 2018). This has led to a much more nuanced understanding of the genes involved and a marked expansion in the number of cell death programs associated with neurodegenerative disease. Here, this expansion in molecular understanding enabled us to genetically or chemically modulate key factors in apoptosis, necroptosis and parthanatos. We selected caspase-3 as a key target for apoptotic pathway manipulation, as both the intrinsic and extrinsic apoptotic pathways undergo mitochondrial outer membrane permeabilization (MOMP), resulting in caspase-3 activation (Moujalled et al., 2021; Vitale et al., 2023). In necroptosis, death ligand binding to a receptor enables RIPK1 activation of RIPK3, which then goes on to activate mixed lineage kinase domain like pseudokinase (MLKL), resulting in plasma membrane permeabilization (Balusu and De Strooper, 2024; Guo et al., 2024; Moujalled et al., 2021). As such, we modulated RIPK3 and the zebrafish ortholog of human RIPK1, Ripk1l, to explore the effects of inhibiting the necroptosis pathway. In parthanatos, hyperactivation of poly(ADP-ribose) polymerase 1 (PARP1), a component of the DNA damage response machinery, results in redox collapse and MOMP (Guo et al., 2024; Moujalled et al., 2021; Yu et al., 2002). As PARP1 hyperactivation is a critical step in the initiation of the parthanatos pathway, it was an ideal target for parthanatos inhibition. Although parthanatos is the primary pathway implicated, the rescue seen from *parp1* crispants was relatively modest. This may be due to activity of other PARP enzymes (zebrafish have 17 Parp family members), although PARP1 is the only variant strongly linked to parthanatos, or the possibility that inhibition of one pathway does not preclude stressed cells from shunting down an alternative pathway. Indeed, this is supported by additive or synergistic effects on survival of rod cells following NTR/MTZ ablation when inhibition of multiple pathways with paired drug treatments was performed (Zhang et al., 2021). Furthermore, there were some differences between chemical inhibitors and genetic disruption, as seen by apoptosis inhibition leading to rod rescue in the crispant experiments but not in the inhibitor assays. This could be because of the gene knockdown allowing more time throughout development for compensatory mechanisms to be called into play prior to induction of cell death (5 days versus 4 h for the chemical inhibitors). Additionally, the N-phenylthiourea (PTU) treatment used to block melanophore production and enhance optical clarity of zebrafish larvae has been shown to induce autophagy, a primarily cytoprotective process (Chen et al., 2021). Given the known crosstalk between autophagy and other cell death pathways (Galluzzi et al., 2018; Nikoletopoulou et al., 2013), synergistic or masking effects may occur in our experimental conditions – despite controls and experimental zebrafish both undergoing the same PTU treatment.

All three cell death pathways tested are associated with neurodegenerative disease progression (Guo et al., 2024; Moujalled et al., 2021) in patients and animal disease models (Park et al., 2020; Thal et al., 2024; Vitale et al., 2023). For instance, modulation of apoptotic factors can improve neuron survival in animal models of PD (Vitale et al., 2023), amyotrophic lateral sclerosis (ALS) (Vitale et al., 2023) and glaucoma (McKinnon et al., 2002), and knockdown or inhibition of necroptosis pathway components can ameliorate neuron loss seen in models of AD (Balusu and De Strooper, 2024), PD (Zhang et al., 2017), ALS (Thal et al., 2024), ischemia reperfusion (IR) (Rosenbaum et al., 2010) and secondary cone photoreceptor loss seen in RP (Murakami et al., 2012). Similarly, poly(ADP-ribosyl)ation was found in brain sections from patients with AD, PD and ALS (Curtin and Szabo, 2013). Chemical or genetic inhibition of parthanatos has been used to protect neurons in animal models of AD (Park et al., 2020), PD (Curtin and Szabo, 2013; Koh et al., 2005), RP (Paquet-Durand et al., 2007; Sahaboglu et al., 2010) and IR (Dvoriantchikova et al., 2022; Yang and Sun, 2023). Here, we implicate parthanatos as central to retinal neuron loss in the NTR 2.0/MTZ ablation paradigm. Given these findings, the NTR/MTZ system may not be an ideal method of modeling diseases in which other cell death pathways are implicated in cell loss but could be a powerful resource for modeling diseases such as RP, PD and other conditions in which parthanatos has been implicated.

Interestingly, there is evidence that the type of cell death elicited during neurodegenerative disease can differ across cell types (Thal et al., 2024). For instance, in AD, proteins indicative of necroptosis are observed in neurons (Koper et al., 2020), but pyroptosis is implicated in the loss of microglial cells and astrocytes (Moonen et al., 2023). In ALS, oligodendrocytes are susceptible to necroptosis, but motor neurons are seemingly not (Ito et al., 2016). These differences are not limited to neurons versus glia. Even different types of neurons have been shown to have different responses. For instance, in the rd10 mouse model, rods die by parthanatos but cones display necrotic morphology and can be rescued by RIP3 deficiency or RIPK inhibitor treatment (Murakami et al., 2012). However, it is unclear whether this is due to differences in cell types versus in the timing or rate of cell loss (e.g. rod death is observed much sooner than secondary cell loss despite the same degenerating environment). Furthermore, whether cell death pathway specificity extends to cell subtypes is still being explored. These and related studies inspired us to investigate NTR 2.0/MTZ-mediated mechanisms of death from a cell type-specific perspective. Our findings highlight the need for increased understanding of the cell death pathways underlying neuronal loss in neurodegenerative diseases and in our disease models. Collectively, these data suggest that targeting parthanatos should improve cell survival but also suggest that a combinatorial approach targeting multiple cell death pathways may further improve therapeutic effects for patients.

### The NTR 2.0/MTZ system can be used as an inducible model of neurodegenerative disease

Mutational neurodegenerative models can provide insight into the cell death mechanisms underlying neuronal cell loss as well as insights into mechanisms leading to disease onset and progression (Dawson et al., 2018). Inducible disease models generally fail to provide mechanistic disease insights but, nevertheless, can be useful. For instance, by providing control over the onset of cell loss, inducible models such as the NTR/MTZ-based cell ablation system simplify the process of screening for neuroprotective factors. Accordingly, we established a robotics-automated platform enabling large-scale drug discovery directly in inducible degenerative disease models – e.g. zebrafish or hiPSC-derived organoids – in which targeted cell types express a reporter enabling quantification of cell number (Vergara et al., 2017; White et al., 2016; Zhang et al., 2021). In addition, inducible systems are titratable, which allows control over the rate of cell death, facilitating investigations into how the extent of cell loss impacts disease-relevant aspects of disease, such as immune system reactivity. Similarly, methods enabling sustained cell loss, such as the improved NTR 2.0 system, allow inroads into how chronic inflammation impacts other disease-relevant parameters, such as the ability to regenerate lost neurons in species such as zebrafish. Owing to these advantages, and the insights provided here about NTR/MTZ-mediated cell death, the NTR 2.0 system can be viewed as a viable approach for modeling degenerative disease.

### NTR-H as a model of neurodegenerative disease in human organoids

Human organoids provide useful tools to study and model neurodegenerative and retinal degenerative diseases, but they have mainly relied on exposure to hypoxia, oxidative stress or mutations to induce a degeneration phenotype (Artero Castro et al., 2019; Ashworth et al., 2024; Kim et al., 2021; Oyefeso et al., 2021). In this study, we proposed a new model system of neurodegenerative disease in human tissues by combining hiPSC-derived retinal organoids with NTR-H/MTZ cell ablation technology. First, we demonstrated that the new NTR-H variant identified here, derived from *C. sakazakii*, showed cell ablation efficiency in hiPSC- and iPSC-derived retinas. We then highlighted the applicability of this system for disease modeling by biochemically characterizing the cell death mechanisms triggered in this paradigm. In accordance with our zebrafish data, our results also show proof of principle that the NTR-MTZ system can be used to induce pathologically relevant forms of cell death, such as parthanatos, in hiPSC-derived retinal organoids. In addition to modeling neurodegenerative disease, retinal organoids have been used to develop and test potential neurodegenerative disease treatments, given that organoids can be easily manipulated in culture, and cellular and molecular assays can be used to analyze any potential rescue or response (Ashworth et al., 2024). Given this, there has been a concerted effort to improve retinal organoid technology to make it amenable to high-throughput preclinical screening (Ashworth et al., 2024; Zhao and Yan, 2024). The NTR/MTZ system is well suited for high-throughput screens given the ability to control onset, duration (Sharrock et al., 2022) and the extent of cell loss, as demonstrated by Zhang et al. (2021) in a large-scale neuroprotectant screen for RP. The advent of NTR-H/MTZ in retinal organoids affords the ability to perform high--throughput phenotypic screens in preclinical neurodegenerative disease models, opening avenues for disease mechanism and treatment exploration.

## MATERIALS AND METHODS

### Ethics statement

The zebrafish protocol was approved by the Animal Care and Use Committees of the Johns Hopkins University School of Medicine. All zebrafish experiments were conducted in accordance with both the Association for Research in Vision and Ophthalmology (ARVO) statement on the ‘Use of Animals in Ophthalmic and Vision Research’ and the National Institutes of Health (NIH) Office of Laboratory Animal Welfare (OLAW) policies regarding studies conducted in vertebrate species. The use of hiPSCs in this study conforms to the University of Colorado Office of Regulatory Compliance.

### Transcriptomic data processing

Previously published data from a RGC ablation paradigm (GSE268179) were reanalyzed (metadata in Dataset 2) for expression of parthanatos, necroptosis and apoptosis. The data were filtered (n_reads>100), and the top 5000 most highly variable genes were selected, normalized and log-plus-1 transformed. Principal component analysis (PCA) was run. Cells were then clustered using the Leiden algorithm on the PCA embedding (Fig. S1A) and annotated using representative sets of marker genes (Dataset 3, Fig. S1B,C). The clusters identified by the Leiden segmentation with overlapping coarse cell type were merged. The transcript counts were pseudobulked by time, experimental condition and coarse cell type.

### Cell death pathway scoring

Next, several publications and datasets (Chen et al., 2026; Fan et al., 2024; Gadepalli et al., 2021; Galluzzi et al., 2012; Huang et al., 2022; Qi et al., 2025; Sun et al., 2024; Wang et al., 2023; Wu et al., 2024; Yao et al., 2024) were used to generate a consensus gene list/signature (Dataset 5) associated with each cell death pathway, and orthologs were mapped to the zebrafish genome. A score for each signature was created (as per Tirosh et al., 2016) using the scanpy implementation. The s.e.m. for each time point is overstated owing to lost annotations for intra-individual variance, so caution is warranted in interpretation. We established a baseline for signature validity by constructing an empirical distribution of the injury-control score difference over 1000 bootstraps of sham signatures of comparable size.

### DEGs and GSEApy of RGC ablation

DEGs were identified globally using PyDESeq2 (Love et al., 2014; Muzellec et al., 2023), fitting a negative binomial generalized linear model (GLM) for ablation, time and cell type (‘∼ exp_condition+cell_type+time’) DEGs were again identified in the context of RGCs alone, fitting a negative binomial GLM for ablation and time. We then assessed pathways enriched in the top 100 upregulated and downregulated DEGs both globally and for RGCs only using GSEApy against the KEGG 2019 zebrafish catalog. DESeq2 used a Wald test (with a Benjamin–Hochberg correction in Dataset 4).

### Zebrafish maintenance and husbandry

Zebrafish (*Danio rerio)* were maintained at 28.5°C with a light cycle of 14 h of light and 10 h of dark. A newly generated transgenic line, *Tg(rho:GAP-YFP-2A-nfsB_Vv F70A/F108Y)jh405* (referred to as *rod:YFP-NTR 2.0*) expresses a membrane tagged YFP and an improved version of NTR exclusively in rod photoreceptors (Sharrock et al., 2022). YFP allows the visualization and quantification of rod cells, and NTR enables induction of cell death in the presence of prodrug – MTZ. This line was propagated in a pigmentation mutant with reduced iridophore numbers, *roy^a9^* (*roy*), to facilitate YFP reporter signal detection *in vivo.* Furthermore, a transgenic line was made that expresses the following two transgenes: *Tg(isl2b:Gal4, myl:RFP)* and *Tg(UAS: YFP-P2A-nfsB_Vv F70A/F108Y)* (*RGC:YFP-NTR 2.0*). This line expresses a membrane-tagged YFP and the improved version of NTR exclusively in RGCs (Emmerich et al., 2024). In these fish, YFP allows the visualization and quantification of RGCs, and the NTR enables induction of cell death upon the presence of MTZ.

### Rod and RGC loss model and YFP quantification analysis

Zebrafish *rod:YFP-NTR 2.0* embryos and *RGC:YFP-NTR 2.0* embryos were collected. At 16 h post fertilization (hpf), embryos were treated with N-phenylthiourea (PTU; Tokyo Chemical Industry P0237) added to E3 medium (E3/PTU) to achieve a final concentration of 0.2 mM to suppress melanophore maturation, promoting ocular transparency and facilitating YFP imaging and quantification. The larvae were divided into six groups with 12 to 24 larvae per group at 5 dpf. Each group was treated with MTZ (ACROS ORGANICS 210341000 for rods and RGCs; Thermo Fisher Scientific 210341000 for RGCs) at various concentrations (50, 25, 12.5, 6.25, 3.125 and 0 μM) for 2 days. To minimize light-induced startle responses (Walker et al., 2012; White et al., 2016), larvae were anesthetized with 1× clove oil (Sigma-Aldrich E51791-100G) and underwent automated reporter quantification *in vivo* (ARQiv) assay (Walker et al., 2012; White et al., 2016). YFP signal quantification was performed using a fluorescence microplate reader (TECAN Infinite M1000 PRO; excitation, 508 nm (rods) or 511 nm (RGCs); emission, 528 nm, bandwidth, 5 nm). Non-transgenic *roy* fish were used to define background signal cutoff values for rod experiments, and fish with only red heart tracers (*isl2b:Gal4;myl:RFP*) were used to define background signal cutoff values for RGC experiments. All collected larvae were randomly divided into several groups to receive treatment/injection. To ensure adequate statistical power, data from three to four experiments were pooled to achieve a sufficiently large sample size per group. Data from each experiment were normalized to untreated controls, then combined to derive the final result.

### Confocal imaging of zebrafish

Zebrafish *rod:YFP-NTR 2.0* and *RGC: YFP-NTR 2.0 l*arvae were anesthetized in 0.016% tricaine (Syndel Syncaine MS-222) and embedded in 1% low-melt agarose gel (Fisher Scientific BP1360-100). An Olympus Fluoview 1000 confocal microscope with a 4× air objective was used to image the whole fish, and a 20× water immersion objective (0.95 NA) was used to collect 30- to 40-slice *z*-stack images of YFP-expressing cells across the whole retina at 4 μm intervals for rods and 5 μm intervals for RGCs. These images were *z*-stacked into single images using maximal intensity projection method in ImageJ. Images in Fig. 2E were stacked into single images using standard deviation projection to minimize saturation.

### Cell death pathway inhibitor assays

To evaluate the cell death pathways implicated in MTZ-NTR 2.0-induced rod photoreceptor and RGC death, multiple cell death inhibitors – including eight parthanatos inhibitors, two necroptosis inhibitors and three apoptosis inhibitors – were selected to test for the neuroprotective effect (Fig. S1B; Dataset 1). The applied concentrations of all parthanatos inhibitors and apoptosis inhibitors were referenced to our previously published results (Zhang et al., 2021). The concentrations of two necroptosis inhibitors were determined by referencing the drug titration results in *rod:YFP-NTR 1.0* larvae. GSK963 was tested from 64 to 0.125 μM, and RIPK3 inhibitor was tested from 16 to 0.25 μM, with a twofold dilution. *YFP-NTR2.0*-positive fish were pretreated for 4 h with 1.5-2× solutions. Addition of MTZ resulted in 1× MTZ and chemical inhibitor dilution to 1× and was used for the 48 h treatment. As drugs were in 0.1% DMSO, all controls (no MTZ, +MTZ and background cutoff) were kept in 0.1% DMSO. YFP signal levels were measured by ARQiv assay. Sample size was determined in accordance with our previous large-scale chemical screen (Zhang et al., 2021). To combine and analyze the data from different experiments, data normalization was conducted by (*S*_i_)/(_Neg_), where ‘*S*_i_’ is the signal of the i^th^ reading, and ‘_Neg_’ is the mean of the negative controls. *P*-values were calculated by Kruskal–Wallis with Dunnett’s post-hoc analysis.

### CRISPR/Cas9-based gene knockdown

To further assess which cell death pathway is involved in NTR 2.0/MTZ cell-specific ablation, key genes for each cell death signaling – *parp1* for parthanatos, *ripk1l* for necroptosis, *casp3a* and *casp3b* for apoptosis – were selected for knockdown by CRISPR/Cas9-based ‘redundant targeting’ methodology (Wu et al., 2018; Zhang et al., 2021). Two sgRNAs were designed following guidelines from Lin et al. (2025). Briefly, sgRNAs were designed using a combination of CHOPCHOP, CRISPOR and the UCSC Genome Browser, prioritizing guides that cut in N-term of protein domains (UniProt annotated) and have Lindel scores >75. Among candidates, guides that have higher CCTop scores, reduced off-targets and high specificity scores were prioritized. Additionally, sgRNAs beginning with GN or NG were prioritized to facilitate T7 *in vitro* transcription. To validate our studies and account for off-target effects, four additional sgRNAs targeting each gene and non-overlapping with the two sgRNAs previously tested were either selected from the dataset generated by Wu et al. (2018), or designed using CRISPRscan. Off targets for all sgRNAs can be found in Dataset 1. Preparation of sgRNAs using the High Scribe T7 *in vitro* transcription kit (New England Biolabs E2040L) followed the published method (Wu et al., 2018) and our previous report (Zhang et al., 2021).

Briefly, zebrafish embryos were collected and divided into two groups – the injected group and the control group. To knock down *parp1* and *ripk1l*, two (or four for validation) sgRNAs (1 μg per μl total) targeting each gene and 5 μM Cas9 protein (20 μM, IDT M0646M) were co-injected into zebrafish embryos at the one-cell stage. Because zebrafish have paralogs *casp3a* and *casp3b*, for knockdown, two (or four for validation) sgRNAs targeting each gene (four sgRNAs, eight for validation) were pooled and co-injected with Cas9 protein into zebrafish embryos. All embryos were PTU treated to suppress black pigmentation at 16 hpf. Each group, the crispant and control, was further divided into two subgroups, and, on 5 dpf, the groups were treated with and without the concentration of MTZ that will enable 50% ablation at 6 dpf. Rod cell and RGC survival were evaluated by quantifying rod-YFP and RGC-YFP using ARQiv assay at 6 dpf. The reading of MTZ-treated embryos was first normalized to scramble unablated control (Fig. 3; uninjected unablated for Fig. S3) or to the unablated control in the same crispant group (Fig. 5; Fig. S4). The ratio of MTZ-induced cell death was compared between injected and control groups. The levels of rod-YFP and, subsequently, the RGC-YFP of the injected and control groups without MTZ ablation were compared separately to evaluate the influence of microinjection. After checking for normality, Kruskal–Wallis with Dunnett's post-hoc analysis was used. Three to four biological repeats were performed.

### Quantitative PCR

To confirm the CRISPR/Cas9-based gene knockdown effect, 11-20 larvae without MTZ ablation from the injected and control group were pooled respectively for mRNA extraction at 6 dpf. All RNAs were purified using a Monarch RNA Cleanup kit (NEB T2040L) and reverse transcribed using a qScript cDNA synthesis kit (QuantaBio 95047-100) following the manufacturers’ instructions. Quantitative PCR was performed using published primers (Zhang et al., 2021; Dataset 1) and PowerUp™ SYBR™ Green Master Mix (ABI) in QuantaStudio (ABI). Delta delta CT analysis was conducted to compare the gene expression levels between injected and control larvae, using *actb1* and *rplp0* as reference genes. Each experiment was performed in triplicate or quadruplicate. All quantitative PCR data including C_t_ readings can be found in Datasets 8 and 9.

### Generation of transgenic hiPSC lines

The episomal hiPSC line derived from CD341 cord blood (Thermo Fisher Scientific A18945; Burridge et al., 2011) was used to generate transgenic hiPSC lines. The cell line was obtained with verified normal karyotype and was analyzed for pluripotency, bacterial, fungal and mycoplasma contamination. Additionally, hiPSC-derived retinal organoids were routinely retested for mycoplasma contamination by PCR. Transgenic cell lines were generated by electroporation with a Neon Transfection System (Invitrogen NEON1SK) according to the manufacturer’s instructions, as described in Ranganathan et al. (2014). The PiggyBac transposon-mediated constitutively expressed membrane YFP hiPSC line was generated using 1 μg PB-myr-tagged YFP donor vector, 0.3 μg PB-HA transposase expression vector (Wellcome Trust Sanger Institute; (Cadiñanos and Bradley, 2007) and 1 μg NTR vector (NCBI non-redundant protein ID WP_012124808). Briefly, cells were pre-treated with 5 μM blebbistatin (Sigma-Aldrich B0560-5MG) for 24 h to increase cell viability, followed by treatment with Accutase (Stemcell Technologies 07920) for 5 min, dissociation into single cells, centrifugation at 80 ***g*** for 5 min for pellet formation and incubation on ice for 15 min. The plasmid was combined in R buffer (Invitrogen N1025), resuspended in the plasmid cocktail (see above) and electroporated with a 10 μl tip-type and the following parameters: 1300 V; 20 ms pulse length; one pulse. Cells were then gently resuspended into 1 ml mTeSR1 plus 5 μM blebbistatin, incubated at room temperature for 20 min, and plated onto Matrigel-coated 35 mm TC-treated dishes containing mTeSR1 (Invitrogen 05850) and 5 μM blebbistatin. Finally, cells were incubated at room temperature for 20 min and cultured thereafter at 37°C and 5% CO_2_. After 5 days, a single clonal subline was expanded and used for all downstream retinal organoid differentiations and MTZ experiments. Transgene copy number was not directly quantified. For the comparison between WT, YFP-NTR-H and chimeric organoids (Fig. S5), WT organoids do not contain YFP and did not contain piggyBac. Any detected YFP is background or intrinsic autofluorescence.

### NTR variant analysis in *E. coli* cells

To assess MTZ reductase activity in a minimal nitroreductase background, the *E. coli* 7NT strain was used, which bears in-frame deletions of seven candidate NTR genes (*nfsA*, *nfsB*, *azoR*, *nemA*, *yieF*, *ycaK* and *mdaB*) and the efflux pump gene *tolC* (Copp et al., 2017). Day cultures of strains overexpressing candidate NTR genes from plasmid pUCX (Addgene plasmid #60681) from a previously established collection (Copp et al., 2017) were set up by adding 150 μl overnight culture to 3 ml fresh lysogeny broth (LB) amended with 100 μg/ml ampicillin and 50 μM IPTG in a 15 ml tube, for each strain. Protein expression was induced in fresh day cultures for 2 h at 30°C with shaking at 200 rpm. For growth inhibition assays, 40 μl culture aliquots were added to 384-well plate individual wells containing 40 μl LB, either amended with MTZ at twice the desired final concentration or unamended as an unchallenged control. Culture turbidity [optical density at 600 nm (OD_600_)] was recorded initially (*T*_0_) and following 4 h incubation at 30°C, 200 rpm (*T*_4_). Percentage growth was then calculated by subtracting the *T*_0_ value from the *T*_4_ for each well, then dividing the data for challenged wells by the corresponding unchallenged replicate. Half-maximal inhibitory concentration (IC_50_) assays used increasing titrations of MTZ (nine conditions containing twofold stepwise increases) and one induction medium-only control. IC_50_ values for each strain were calculated using a dose-response inhibition four-parameter variable slope equation in GraphPad Prism 7.0 (GraphPad Software Inc.).

### NTR variant selection in hiPSC

Transgenic hiPSC were constructed to express a P2A polycistronic vector with YFP and the NTR variant previously identified by Copp et al. (2017) – NfsB_Cs. This NTR variant was preselected using the same preselection methodology used in Sharrock et al. (2022). The resulting hiPSC were co-cultured with WT hiPSC in Matrigel (growth factor reduced; Corning 354230)-coated 24-well plates with mTEsR1 medium (Stemcell Technologies 05850). MTZ was added to the medium at 0, 0.1, 0.5, 1, 5, 10 and 20 μM final concentration and maintained for 24 h. Medium was then replaced with mTeSR1, and plates were imaged using a Cellomics ArrayScan VTI HCS platform. Experiments were performed in triplicates, and the numbers of transgenic and WT colonies were quantified.

### hiPSC-derived retinal organoid culture

Three-dimensional retinal organoids were generated from hiPSCs as previously described (Zhong et al., 2014) Succinctly, hiPSC were maintained on Matrigel (growth-factor-reduced; BD Biosciences)-coated plates with mTeSR1 medium. On day (D)0 of differentiation, hiPSC colonies were treated with Dispase 1 U ml^−1^ in DMEM/F-12 (Stem Cell Technologies 07923), mechanically dissociated into small clumps and cultured in suspension to induce neural aggregate formation. On D1, aggregates were gradually transitioned into a neural-induction medium [prepared by combining 489 ml DMEM/F12 (1:1; Life Technologies 11330-057) with 1 ml heparin (1 mg/ml stock in DMEM, 0.1%; Sigma-Aldrich H3149-100), 5 ml MEM Non-Essential Amino Acids (100×; Life Technologies 11140-050), and 5 ml N2 Supplement (100×; Life Technologies 17502-048)] to induce anterior neural differentiation. On D7, neural aggregates were seeded onto Matrigel-coated dishes and cultured in retinal differentiation medium (RDM; prepared by combining 320 ml DMEM (Life Technologies 11965) and 160 ml F12 (Life Technologies 11765) with 5 ml Antibiotic–Antimycotic (100×; Life Technologies 15240), 5 ml MEM Non-Essential Amino Acids (100×; Life Technologies 11140-050), and 10 ml B27 Supplement without vitamin A (50×; Life Technologies 12587-010). Between D16-D22 of differentiation, horseshoe-shaped neural retina (NR) domains were mechanically detached and cultured in suspension in RDM, in which three-dimensional retinal organoids gradually formed. For long-term suspension culture, the medium was supplemented with 10% fetal bovine serum (R&D Systems S11150), 100 μM taurine (Sigma-Aldrich T0625) and 2 mM GlutaMAX (Invitrogen 35050061) beginning on D42, with the addition of 1 mM all-trans retinoic acid (Sigma-Aldrich R2625-50MG) during week 10-14 and 0.5 mM retinoic acid thereafter. All the cultures from hiPSC to retinal organoids were performed at 37°C in a humidified 5% CO_2_ incubator. Where indicated, total YFP fluorescence intensity in live organoids at 80 days of differentiation was quantified using a TECAN Infinite M1000 microplate reader as previously described (Vergara et al., 2017).

### MTZ treatment of retinal organoids and cell death evaluation

To assess MTZ toxicity, WT hiPSC-derived retinal organoids at 80 days of differentiation were incubated with 0, 0.1, 0.5, 1, 5, 10 or 20 μM MTZ in reverse osmosis medium for 24 h at 37°C in a humidified 5% CO_2_ incubator. Cultures were then washed with medium and incubated with the cell-impermeant viability indicator ethidium homodimer-1 (Invitrogen E1169) diluted in 2 μl ml^−1^ medium, for 90 min at 37°C. They were then washed three times in clear RDM and individually plated in black, V-bottom 96-well plates (Corning 3894). Fluorescence intensity quantification was performed using a TECAN Infinite M1000 microplate reader as previously described (Vergara et al., 2017). Parameters used were as follows: excitation wavelength, 525 nm; emission wavelength, 620 nm; bandwidth, 5 nm (excitation/emission); 50 flashes. *N*=3 organoids/condition. Two-way ANOVA with Šídák's multiple comparisons correction was used across time for WT versus NTR-expressing organoids. Kruskal–Wallis test with Dunnett's multiple comparisons correction to the 0 μM MTZ condition was done for comparing the effect of different MTZ concentrations in NTR-expressing organoids.

### Protein extraction and quantification

Three independent experiments were performed for all treatment conditions for protein collection. A minimum of five retinal organoids per sample were collected at day 80 of differentiation, washed in 1× PBS and flash frozen. Proteins were extracted with RIPA buffer (Sigma-Aldrich R0278) containing 1× protease inhibitors (Thermo Fisher Scientific 1860932). After manual homogenization, the samples were kept for 20 min on ice and then centrifuged at 15,000 ***g***, 4°C for 20 min. For western blot analysis, 20 μg (for anti-Caspase antibodies) or 40 μg (for human anti-PAR antibody) of extracted proteins were run on 4-12% NuPage Bis-Tris precast gels (Thermo Fisher Scientific). Proteins were transferred with the semi-dry iBlot 2 Gel transfer device (Thermo Fisher Scientific) to a nitrocellulose membrane. After blocking in 5% low-fat milk 0.1% Tris-buffered saline with 0.1% Tween-20 (TBS-T), membranes were incubated overnight at 4°C with primary antibodies diluted in 5% low-fat milk 0.1% TBS-T. Antibodies used in this study were as follows: anti-Caspase-3 (Cell Signaling Technology 9662) at 1:1000, anti-Cleaved Caspase-3 (Cell Signaling Technology 9664) at 1:1000, anti-human PAR (Kam et al., 2018) at 1:1000 and anti-β-tubulin 50-55 kDa Tubb (Developmental Studies Hybridoma Bank E7) at 1:2000. After three washes in 0.1% TBS-T, membranes were incubated with horseradish peroxidase conjugated goat anti-rabbit IgG (Vector Laboratories PI-1000; 1:2000), horse anti-mouse IgG (Vector Laboratories PI-2000; 1:2000) or anti-human IgG (Abcam ab87422; 1:10,000) antibodies in 5% low-fat milk 0.1% TBS-T (see Dataset 1 for more details). Membranes were then washed in 0.1% TBS-T and developed using SuperSignal West Pico PLUS Chemiluminescent Substrate or SuperSignal West Femto Maximum Sensitivity Substrate (Thermo Fisher Scientific). For Fig. 6J, cell lysates were homogenized in ice-cold RIPA lysis buffer containing 50 mM Tris-HCl (pH 7.4), 150 mM NaCl, 1 mM EDTA, 1% Triton X-100, 0.5% SDS, 0.5% sodium deoxycholate, phosphatase inhibitor cocktails I and II, and a complete protease inhibitor cocktail (Roche 11836170001). Tissue lysates were incubated with gentle rotation at 4°C for 30 min to ensure complete protein extraction, followed by centrifugation at 15,000 ***g*** for 30 min at 4°C. Protein concentrations were quantified using a bicinchoninic acid (BCA) protein assay (Pierce A65453). 20 μg of protein was resolved by SDS-PAGE and transferred onto nitrocellulose membranes (Bio-Rad 1620112). Membranes were blocked with 5% nonfat dry milk in TBS containing 0.1% Tween-20 (TBS-T) for 1 h at room temperature and subsequently incubated with primary antibodies. The antibodies used were as follows: anti-AIFM1 (Santa Cruz Biotechnology sc-13116) at 1:1000, anti-MIF (Abcam ab7207) at 1:1000, anti-LMNA (lamin A/C; Santa Cruz Biotechnology sc7293) at 1:1000, and anti-TUBA1B (Cell Signaling Technology 12351) at 1:5000. After washing, membranes were incubated with species-specific HRP-conjugated secondary antibodies (Cell Signaling Technology 7076 and 7074) at 1:10,000, and immunoreactive bands were detected using enhanced chemiluminescence (same as above). Quantification was performed using Fiji, and protein bands were normalized to the loading control as described in Cañada-García and Arévalo (2023) and Gassmann et al. (2009). Two-way ANOVA with uncorrected Fisher's LSD with a single pooled variance was used to compare groups (*P≤0.05, **P≤0.01, ***P≤0.001, ****P≤0.0001).

### Artificial intelligence tools

No artificial intelligence tools were used in the preparation of this article.

## Supplementary Material

10.1242/dmm.052747_sup1 Supplementary information

Dataset 1. Transgenic resources, chemical inhibitors, primers, sgRNAs, and antibodies.

Dataset 2. Metadata for GSE268179.

Dataset 3. List of genes used to call cell clusters.

Dataset 4. DEGs for RGCs from DESeq2. DEGs generated with DESeq2 between ablated and unablated conditions during RGC ablation made only with the RGC cluster. Includes fold change, unadjusted p-value and adjusted p-value. Positive direction is upregulated in the ablated condition.

Dataset 5. Cell death signatures list. The collection of genes used for the cell death scoring.

Dataset 6. Zebrafish data and summary statistics.

Dataset 7. Zebrafish statistical and normality test results.

Dataset 8. Zebrafish qPCR data and analysis for 2×sgRNAs.

Dataset 9. Zebrafish qPCR data and analysis for 4×sgRNAs.

Dataset 10. Organoid data and summary statistics.
