# Supplementary material for: Cell death analysis of inducible, titratable neurodegenerative disease models in zebrafish and human stem cell-derived retinal organoids
Source: Dis Model Mech. 2026 Jul 24;19(7):dmm052747. doi: 10.1242/dmm.052747 (PMC13474575; doi:10.1242/dmm.052747)
Supplement: Supplementary file 1 [file dmm-19-052747-s1.pdf]

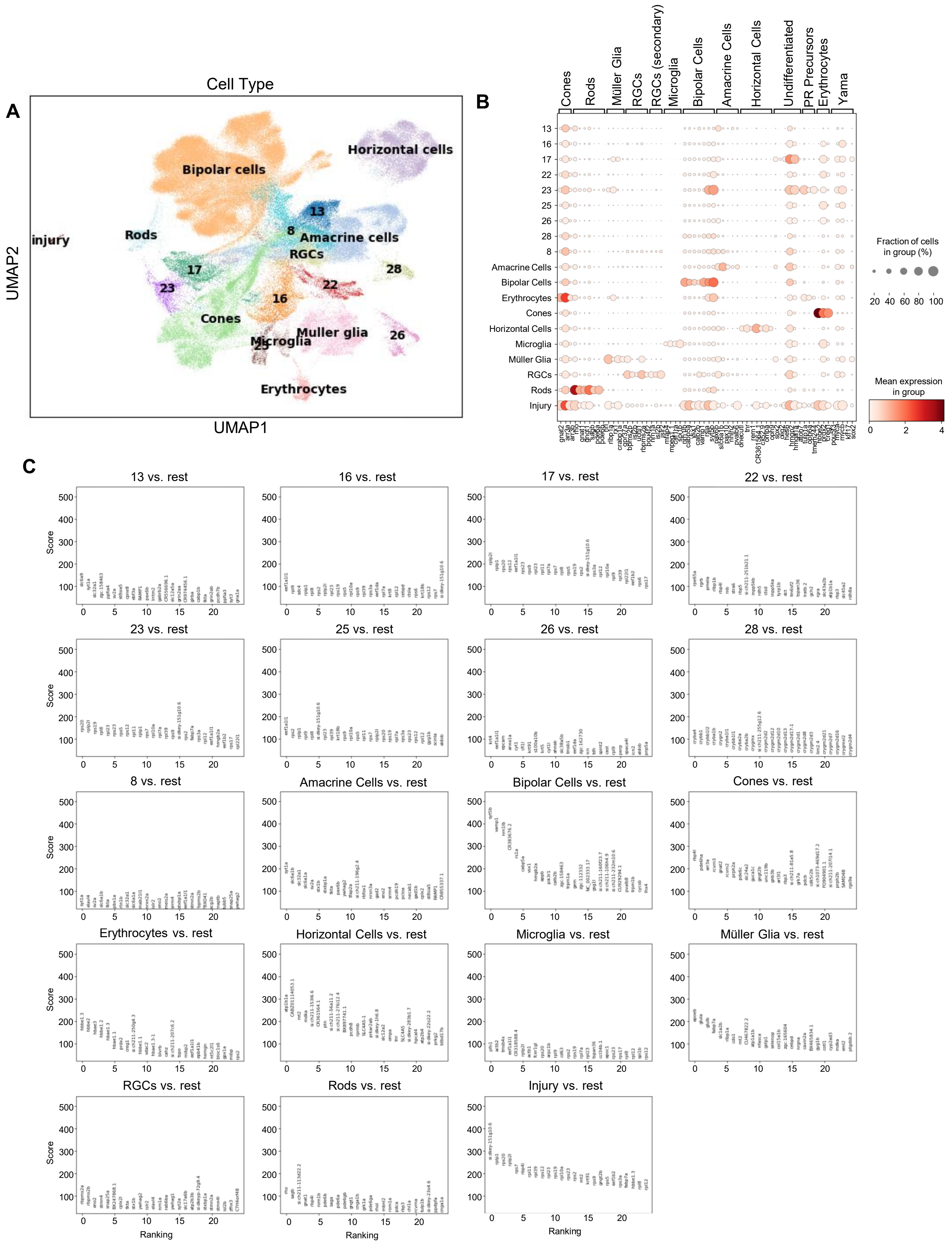

Fig. S1. Transcriptomic delineation of RGCs using Leiden clustering and key gene markers.

A) Annotated UMAP derived from PCA coordinates, colored based on Leiden clustering of all timepoints of the RGC ablation paradigm.

B) Dot plot of key marker genes used to help annotate cell clusters.

C) Top 25 loadings from each leiden cluster used to help annotate cell clusters.

Disease Models & Mechanisms • Supplementary information

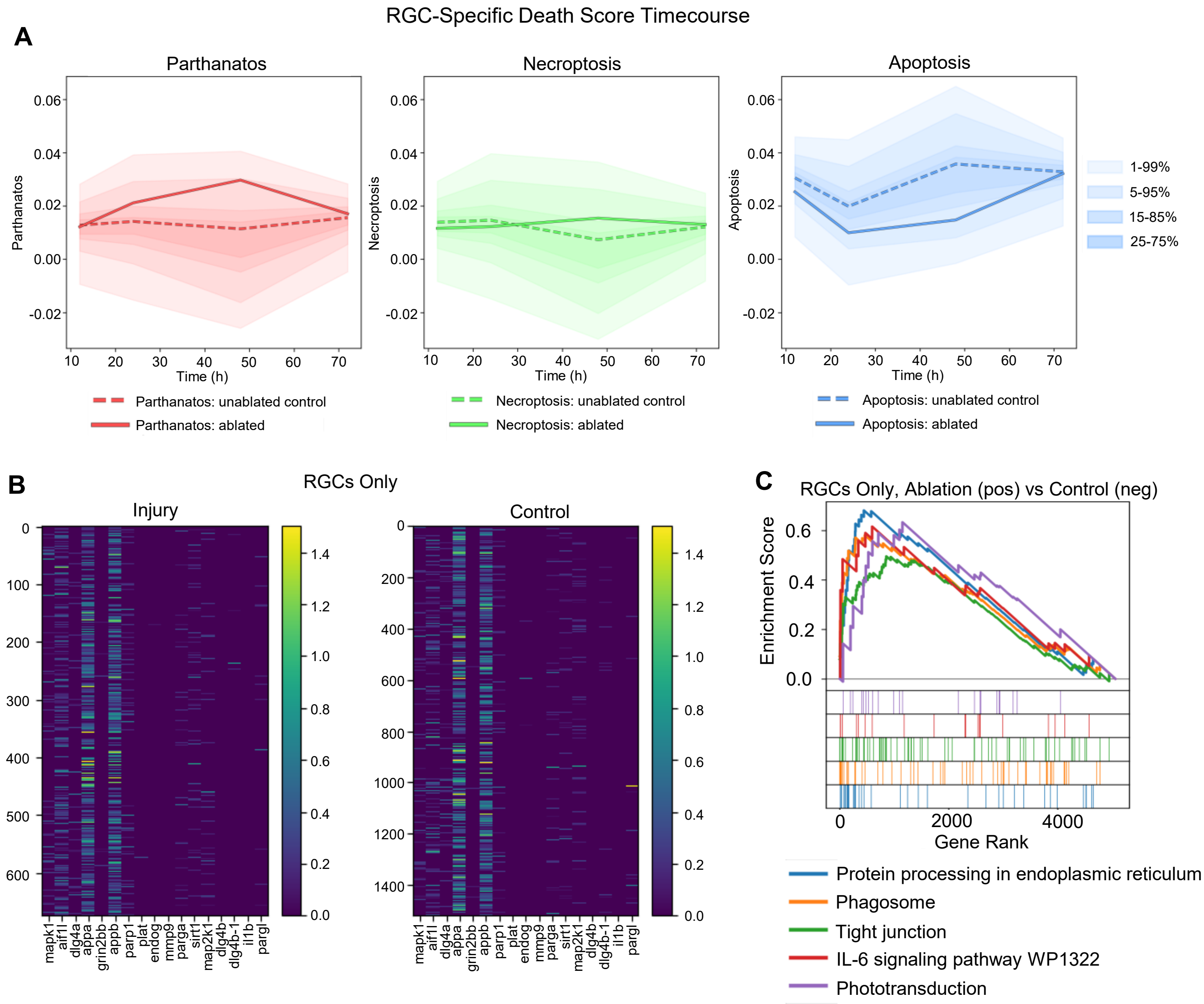

Fig. S2. Cell death pathway analysis of MTZ-treated NTR2-expressing RGCs.

A) Cell Death Pathway Scoring of parthanatos, necroptosis, and apoptosis in RGCs over time. Bold line is the median with a bootstrapped confidence interval notated.

B) Parthanatos-enriched gene expression across all RGCs throughout time in the unablated and ablated conditions.

C) Top: gene set enrichment analysis score for genes differentially expressed between ablated and unablated RGCs, positive score signifies enrichment in the ablated population. Bottom: All genes in the GO gene set ranked by their differential expression between ablated and unablated RGCs.

Disease Models & Mechanisms • Supplementary information

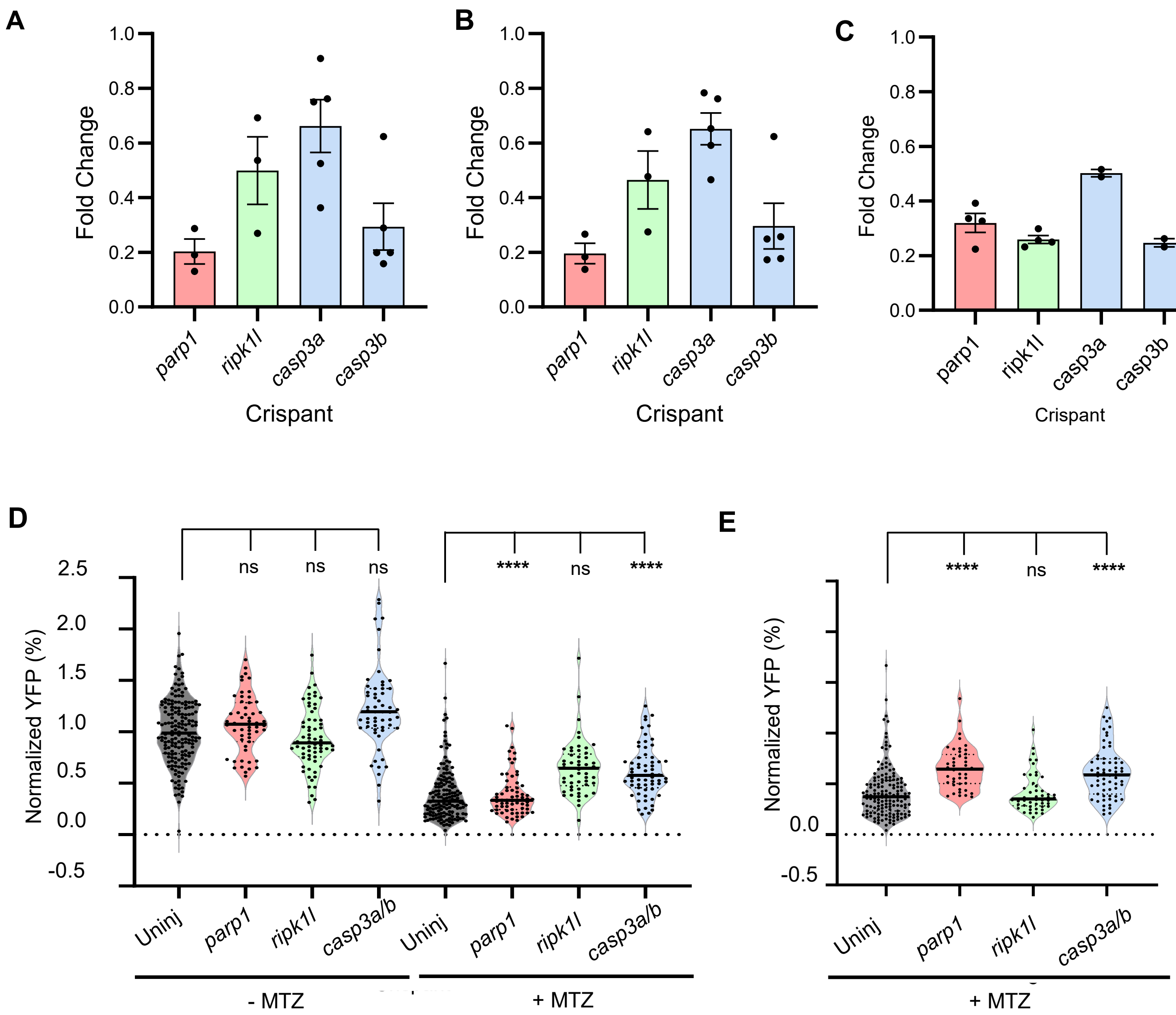

Fig. S3. Validation of crispant studies in MTZ-treated NTR2.0-expressing rods.

A) Quantitative PCR of the 2x sgRNA redundant targeting crispant set depicting target gene expression reduction of *parp1*(red), *ripk1l*(green), and *casp3a/b* (blue) with internal control *rplp0*. Normalized to *scrambled* control. Done in triplicate.

B) Quantitative PCR of the 2x sgRNA redundant targeting crispant set depicting target gene expression reduction of *parp1*, *ripk1l*, and *casp3a/b* with internal control *act1b*. Normalized to *scrambled* control. Done in triplicate.

C) Quantitative PCR of the 4x sgRNA redundant targeting crispant set depicting target gene expression reduction of *parp1*, *ripk1l*, and *casp3a/b* with internal control *rplp0*. Normalized to Uninj Control. Done in triplicate.

D) Effect of 4x sgRNA redundant gene knockdowns on rod cell numbers, obtained via fluorescence quantification, when fish were treated ± MTZ for 24 hrs. Crispants were normalized to the Uninjected No MTZ control. N = 3. One-Way Kruskal-Wallis performed with Dunnett's post-hoc analysis.

E) Cell death response of rods in 4x sgRNA redundant targeting crispants following 24 h treatment with MTZ, each normalized to their respective No MTZ crispant control. One-Way Kruskal-Wallis performed with Dunnett's post-hoc analysis. Table summarizing n, average, and p-value can be found in Datasets 5 and 6.

For all statistical tests, cutoffs are * : P≤0.05; ** : P≤0.01; *** : P≤0.001; **** : P ≤0.0001. Each dot represents an individual larval fish. Gene knockdowns are *parp1*(red), *ripk1l*(green), and *casp3a/b* (blue). For all violins, black line is median. Tables summarizing n, average, and p-value can be found in Datasets 5 and 6.

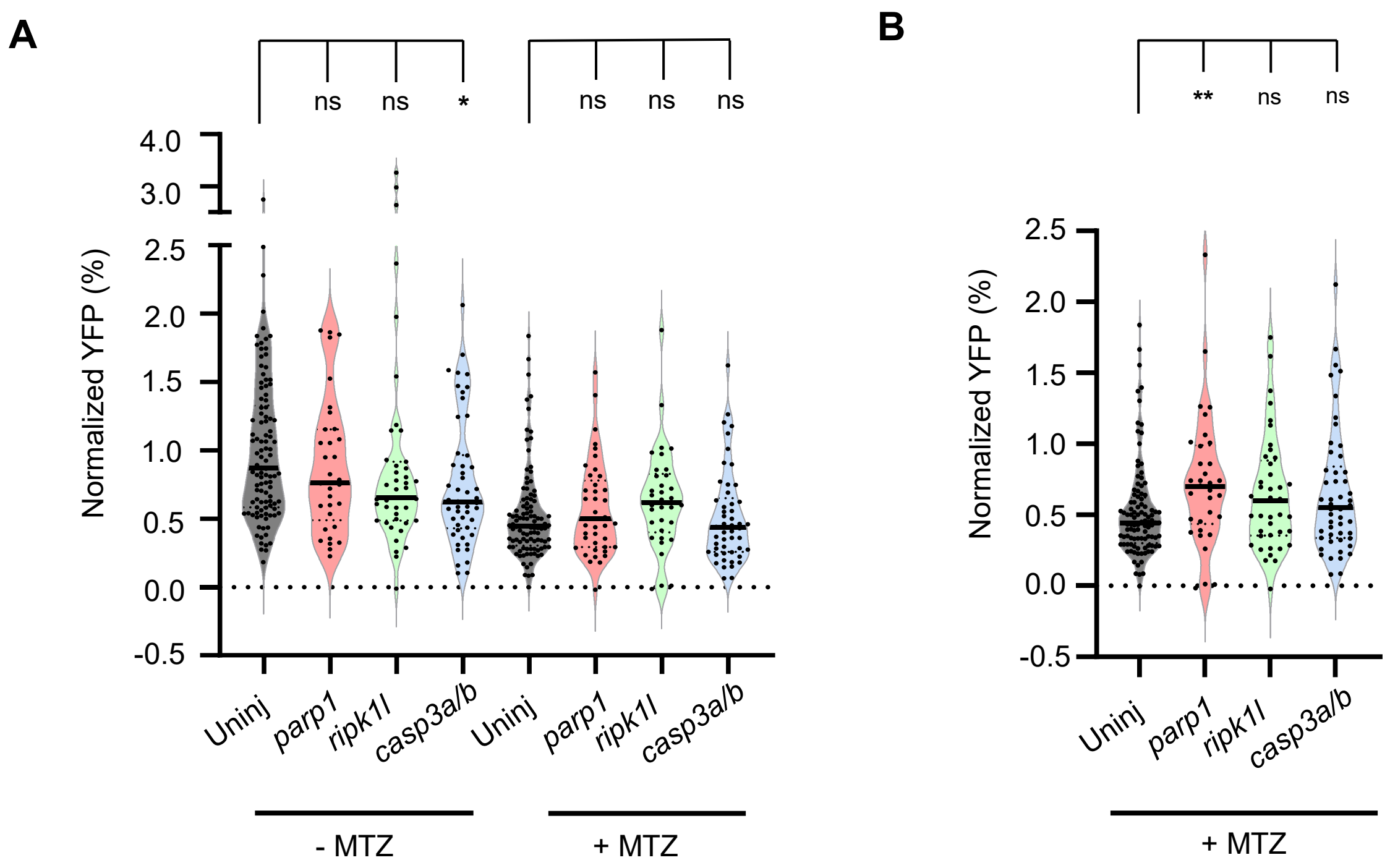

Fig. S4. Validation of crispant studies in MTZ-treated NTR2.0-expressing RGCs.

A) Effect of 4x sgRNA redundant gene knockdowns on RGC numbers, obtained via fluorescence quantification, when fish were treated ± MTZ for 24 hrs. Crispants were normalized to the Uninjected No MTZ control. N = 3. One-Way Kruskal-Wallis performed with Dunnett's post-hoc analysis.

B) Cell death response of RGCs in 4x redundant targeting sgRNAs across three experimental replicates following 24 h treatment with MTZ, each normalized to their respective No MTZ crispant control. One-Way Kruskal-Wallis performed with Dunnett's post-hoc analysis. For all statistical tests, cutoffs are * : P≤0.05; ** : P≤0.01; *** : P≤0.001; **** : P ≤0.0001. Each dot represents an individual larval fish. Gene knockdowns are *parp1*(red), *ripk1l*(green), and *casp3a/b* (blue). For all violins, black line is median. Tables summarizing n, average, and p-value can be found in Datasets 5 and 6.

Disease Models & Mechanisms • Supplementary information

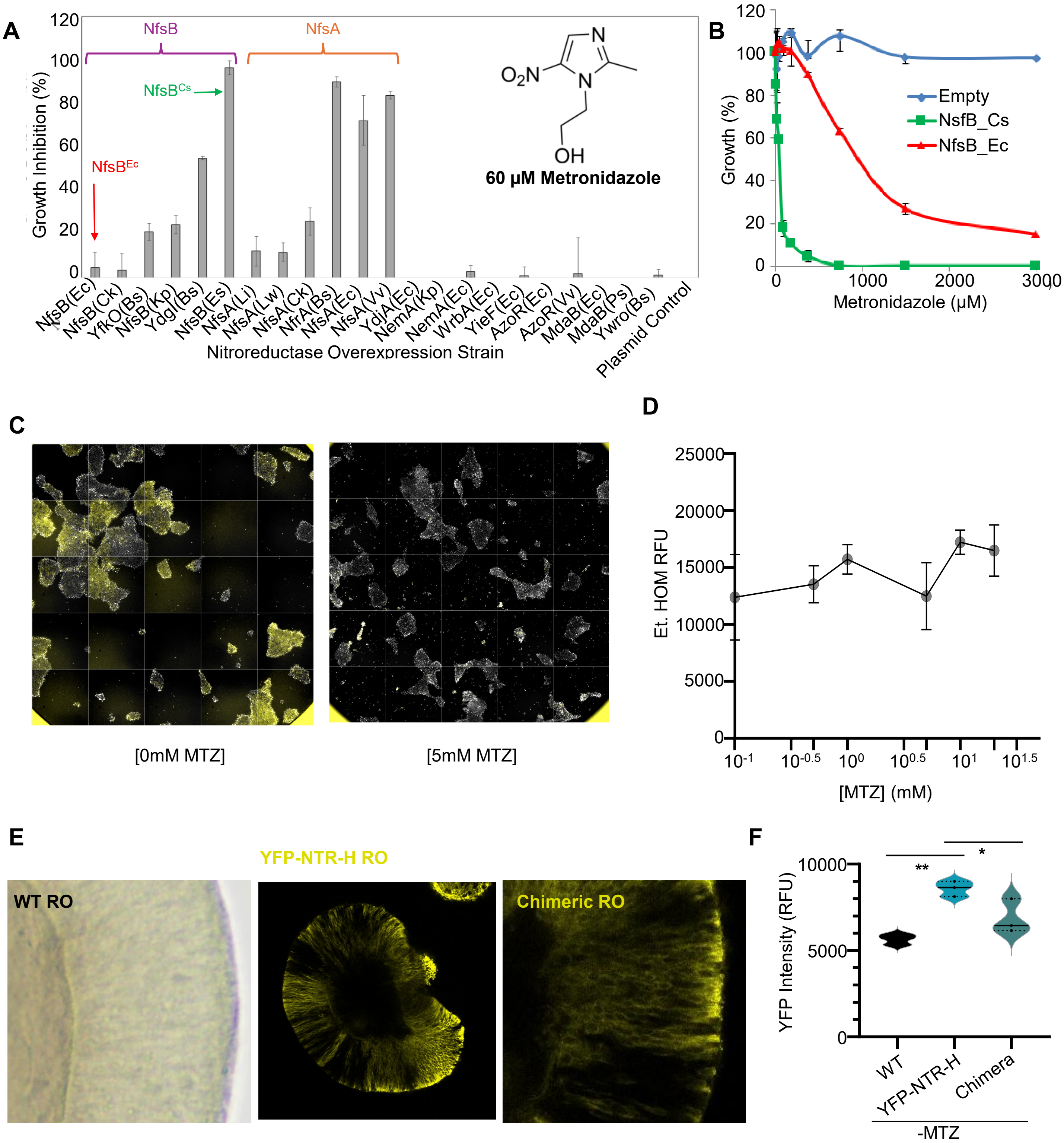

Disease Models & Mechanisms • Supplementary information

Fig. S5. Establishing the MTZ-induced ablation efficiency of NTR-H in retinal organoids.

A) MTZ-mediated growth inhibition of *E. coli* SOS-R2 strains expressing different NTR candidates (as described by Copp et al., 2017). Turbidity (OD_{600}) of NTR over-expressing cell cultures was recorded directly before and after 4 h incubation with 60 µM MTZ. Cell survival is expressed as the percentage decrease in OD_{600} of challenged cells relative to the unchallenged control for each strain post-incubation. Data are the mean (N=2 ± 1 sd) growth inhibition relative to unchallenged cultures. Strains expressing members of the NfsB and NfsA enzyme families are indicated, with NfsB_Ec and NfsB_Cs specifically identified. S5B) Turbidity (OD_{600}) of NTR over-expressing SOS-R2 cell cultures was recorded directly before and after 4 h incubation across a range (0-3.0 mM) of MTZ concentrations. Cell survival is expressed as the percentage decrease in OD_{600} of challenged cells relative to the unchallenged control for each strain post-incubation. Data are the mean (N=2 ± 1 sd) growth inhibition relative to unchallenged cultures. Cells over-expressing NfsB from *C. sakazakii* (NfsB_Cs; IC_{50} 45 µM) were over 20-fold more sensitive to metronidazole than the same cells over-expressing NfsB from *E. coli* (NfsB_Ec; IC_{50} 900 µM).

C) Representative examples of mixed cultures of wild type and transgenic hiPSC for the leading NTR variant identified in our preliminary screening (YFP:NfsB_Cs), treated with 0 or 0.5 mM MTZ respectively. Notice the selective disappearance of YFP(+) colonies upon substrate exposure.

D) WT retinal organoids (RO) were exposed to the same concentration range of MTZ to assess toxicity by ethidium homodimer staining. No statistically significant difference was observed at any of the tested concentrations. N~3/condition. * $p<0.01$. Kruskal-Wallis with Dunn's Multiple Comparisons Test. Mean and SEM displayed. See Supplementary File 9 for more details.

E) Representative images of non-transgenic WT retinal organoids, transgenic retinal organoids expressing YFP-NfsB_Cs (NTR-H), and chimeric retinal organoids generated by mixing WT iPSC with transgenic iPSC at a 65/45 ratio. Scale Bar = 100 µm.

F) YFP fluorescence quantification of live organoids using 3D-ARQ in the absence of a prodrug, to validate the sensitivity of the quantification platform. Ordinary One-Way ANOVA with Tukey's Multiple Comparison Correction.

Disease Models & Mechanisms • Supplementary information

Dataset 1. Transgenic resources, chemical inhibitors, primers, sgRNAs, and antibodies.

Available for download at
https://journals.biologists.com/dmm/article-lookup/doi/10.1242/dmm.052747#supplementary-data

Dataset 2. Metadata for GSE268179.

Available for download at
https://journals.biologists.com/dmm/article-lookup/doi/10.1242/dmm.052747#supplementary-data

Dataset 3. List of genes used to call cell clusters.

Available for download at

https://journals.biologists.com/dmm/article-lookup/doi/10.1242/dmm.052747#supplementary-data

Dataset 4. DEGs for RGCs from DESeq2.

DEGs generated with DESeq2 between ablated and unablated conditions during RGC ablation made only with the RGC cluster. Includes fold change, unadjusted p-value and adjusted p-value. Positive direction is upregulated in the ablated condition.

Available for download at
https://journals.biologists.com/dmm/article-lookup/doi/10.1242/dmm.052747#supplementary-data

Dataset 5. Cell death signatures list. The collection of genes used for the cell death scoring.

Available for download at
https://journals.biologists.com/dmm/article-lookup/doi/10.1242/dmm.052747#supplementary-data

Dataset 6. Zebrafish data and summary statistics.

Available for download at
https://journals.biologists.com/dmm/article-lookup/doi/10.1242/dmm.052747#supplementary-data

Dataset 7. Zebrafish statistical and normality test results.

Available for download at

https://journals.biologists.com/dmm/article-lookup/doi/10.1242/dmm.052747#supplementary-data

Dataset 8. Zebrafish qPCR data and analysis for 2×sgRNAs.

Available for download at
https://journals.biologists.com/dmm/article-lookup/doi/10.1242/dmm.052747#supplementary-data

Dataset 9. Zebrafish qPCR data and analysis for 4×sgRNAs.

Available for download at
https://journals.biologists.com/dmm/article-lookup/doi/10.1242/dmm.052747#supplementary-data

Dataset 10. Organoid data and summary statistics.

Available for download at
https://journals.biologists.com/dmm/article-lookup/doi/10.1242/dmm.052747#supplementary-data
